# Normothermic Perfusion in the Assessment and Preservation of Declined Livers Before Transplantation: Hyperoxia and Vasoplegia—Important Lessons From the First 12 Cases

**DOI:** 10.1097/TP.0000000000001661

**Published:** 2017-01-24

**Authors:** Christopher J.E. Watson, Vasilis Kosmoliaptsis, Lucy V. Randle, Alexander E. Gimson, Rebecca Brais, John R. Klinck, Mazin Hamed, Anastasia Tsyben, Andrew J. Butler

**Affiliations:** ^1^ Department of Surgery, Addenbrooke’s Hospital, University of Cambridge, Cambridge, United Kingdom.; ^2^ The NIHR Cambridge Biomedical Research Centre and the NIHR Blood and Transplant Research Unit in Organ Donation and Transplantation at the University of Cambridge, Cambridge, United Kingdom.; ^3^ Department of Medicine, Addenbrooke’s Hospital, Cambridge, United Kingdom.; ^4^ Department of Pathology, Addenbrooke’s Hospital, Cambridge, United Kingdom.; ^5^ Division of Perioperative Care, Addenbrooke’s Hospital, Cambridge, United Kingdom.; ^6^ University of Cambridge School of Clinical Medicine, Addenbrooke’s Hospital, Cambridge, United Kingdom.

## Abstract

**Background:**

A program of normothermic ex situ liver perfusion (NESLiP) was developed to facilitate better assessment and use of marginal livers, while minimizing cold ischemia.

**Methods:**

Declined marginal livers and those offered for research were evaluated. Normothermic ex situ liver perfusion was performed using an erythrocyte-based perfusate. Viability was assessed with reference to biochemical changes in the perfusate.

**Results:**

Twelve livers (9 donation after circulatory death [DCD] and 3 from brain-dead donors), median Donor Risk Index 2.15, were subjected to NESLiP for a median 284 minutes (range, 122-530 minutes) after an initial cold storage period of 427 minutes (range, 222-877 minutes). The first 6 livers were perfused at high perfusate oxygen tensions, and the subsequent 6 at near-physiologic oxygen tensions. After transplantation, 5 of the first 6 recipients developed postreperfusion syndrome and 4 had sustained vasoplegia; 1 recipient experienced primary nonfunction in conjunction with a difficult explant. The subsequent 6 liver transplants, with livers perfused at lower oxygen tensions, reperfused uneventfully. Three DCD liver recipients developed cholangiopathy, and this was associated with an inability to produce an alkali bile during NESLiP.

**Conclusions:**

Normothermic ex situ liver perfusion enabled assessment and transplantation of 12 livers that may otherwise not have been used. Avoidance of hyperoxia during perfusion may prevent postreperfusion syndrome and vasoplegia, and monitoring biliary pH, rather than absolute bile production, may be important in determining the likelihood of posttransplant cholangiopathy. Normothermic ex situ liver perfusion has the potential to increase liver utilization, but more work is required to define factors predicting good outcomes.

## Abstract 2

This pilot study of normothermic oxygenated perfusion at the end of storage identified optimal oxygenation characteristics associated with improved organ function. Furthermore, bile pH during perfusion was highly predictive of the development of cholangiopathy in DCD donors. These findings will need to be validated with larger sample sizes. Supplemental digital content is available in the text.

The last decade has seen increasing numbers of patients being listed for liver transplantation but a decline in the overall quality of available donor organs, with larger numbers of older donors, donors dying from hypoxic brain injury, and after circulatory death (DCD).^1^ As a consequence in the United States in 2014, 10% of livers recovered from deceased donors were not transplanted in spite of over 3000 potential recipients dying or being removed from the waiting list in the same year.^1^ The situation is similar in the United Kingdom, where 19% of patients either die or are removed from the waiting list within 2 years of listing, whereas 8% of livers recovered from donation after brain death (DBD) donors, and 26% of livers recovered from DCD donors were not transplanted.^2,3^

The principal reason for discard of a liver that has been recovered for transplantation is fear that the liver will not provide life-sustaining function after transplantation, usually in the setting of steatosis, prolonged warm ischemia, adverse hemodynamic characteristics during the DCD withdrawal phase, or prolonged cold ischemia.^4^ Although the liver may have functioned well in the donor, warm and cold ischemia impose an unpredictable injury on the liver that may manifest only after reperfusion in the recipient.

In an effort to increase the utilization of livers, the United Kingdom introduced a “fast-track liver offer scheme” in 1997 to place livers “that have been declined for any reason, or have yet to be accepted, at or after cross-clamp” in the donor.^5^ A simultaneous offer is made to every UK center that has not previously declined the liver. Such livers are typically reported to be abnormal (eg, steatotic, poor in situ perfusion) or were associated with a long withdrawal phase. Because fast-track offering usually takes place after organ recovery, such livers are usually associated with long cold ischemic times. Livers not accepted on the fast-track scheme may be allocated for research if there is appropriate consent. In the period of this report, 587 livers were fast-tracked, with 11% being transplanted.

The ability to undertake normothermic ex situ machine perfusion of livers (NESLiP) has introduced a new dimension to the assessment of donor livers before implantation and enables cold ischemia to be halted while a recipient is prepared for transplantation. Although an initial clinical study has shown encouraging results from normothermic preservation throughout the period of extracorporeal storage,^6^ there are few reports of the use of NESLiP in the assessment of marginal liver grafts.^7^

Against the background of increasingly marginal liver offers, we established a clinical program of NESLiP in our center, targeting livers that were considered potentially viable, but where the ischemic time would be unreasonably long or there was uncertainty about the liver based on the subjective opinion of the retrieving surgeon. The program was paused after 6 cases due to adverse events, and after further investigation and subsequent reduction in the level of oxygenation during perfusion, was restarted. This paper describes our initial experience of 12 cases, and the lessons learned that we believe will benefit all investigators in this area.

## MATERIALS AND METHODS

### Livers and Recipients

Routine national and zonal liver offers, as well as those offered through the UK fast-track liver offering scheme, were considered. Livers that had been offered directly for research were also considered. Normothermic ex situ liver perfusion was typically considered where there was uncertainty about the liver such that the recipient hepatectomy could not start before visualizing the liver, and the prolongation of cold ischemic time that this imposed would have deleterious consequences on the liver. Livers were offered to the highest priority patient (by biochemical or clinical criteria) of suitable size and blood group match who had previously consented for a liver of that type and separately consented for it to undergo NESLiP.

### Preparation

**FIGURE 1:**
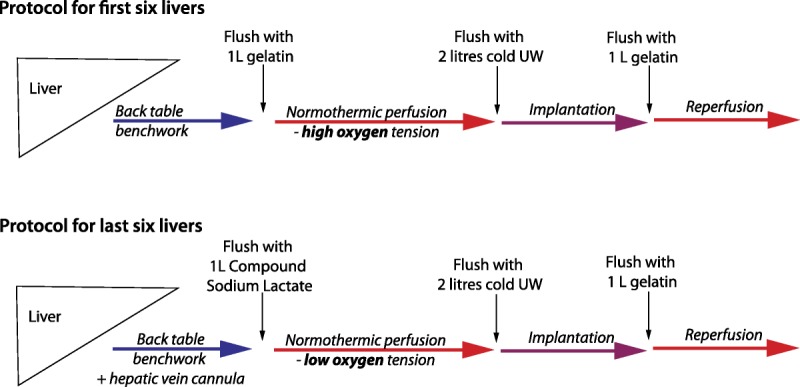
Changes in protocol for normothermic ex situ liver perfusion. Comparison of the protocols for flushing and perfusing the first and last 6 livers undergoing NESLiP.

Livers were prepared for transplantation in a standard manner. In addition, a 6Fr infant feeding tube was placed into the bile duct to drain bile, and the cystic duct was ligated in continuity. Infusion cannulae were tied into the portal vein (PV) and celiac trunk (HA). In the last 6 livers, a 6Fr catheter was also sutured with its tip in the right hepatic vein for direct sampling of venous effluent. The liver was flushed with either a litre of succinylated gelatin (Gelofusine, BBraun Medical, UK) (cases 1 to 6) or with compound sodium lactate (Hartmann solution, Baxter, UK) (cases 7 to 12) at room temperature before NESLiP (Figure 1). Flushing was performed to remove residual UW solution, in particular its potassium. Hartmann solution was used in the last 6 cases to provide lactate as a substrate to enhance monitoring of its clearance during perfusion (1 L contains 29 mmol lactate).

Just before explanting the recipient’s diseased liver, NESLiP was stopped and the donor liver flushed with 2-L ice-cold UW solution (Belzer-UW, Bridge to Life, London, UK). Implantation involved a caval-preserving cavo-cavostomy anastomosis. The liver was flushed with a litre of succinylated gelatin at room temperature before reperfusion via the PV; arterial reperfusion followed portal reperfusion.

### Perfusion Method

Normothermic ex situ liver perfusion was performed using a Liver Assist device (Organ Assist, Groningen, the Netherlands), which provides pressure-regulated flow from 2 independent pumps and oxygenators supplying PV and hepatic artery, respectively. The perfusate comprised 3 units of leucocyte-depleted washed red cells which had a variable volume of around 1 L. The red cells were added to a liter of either succinylated gelatin or Steen solution (Xvivo Perfusion, Göteborg, Sweden) (cases 6 to 8 only) and supplemented with 30 mmol sodium bicarbonate, 25 000 units (50 mg) heparin, antibiotics, calcium chloride, magnesium sulphate, and amino acids (Aminoven-25, Fresenius Kabi Ltd, UK). The final hemoglobin concentration was a median 6.1 g/dL (range, 5.1-7.4); hematocrit, 0.18 (range, 0.16-0.22). Epoprostenol 2 μg/h was given by infusion, and insulin was given either as an infusion or bolus. Bile salts were not administered.

In the first 6 perfusions, the oxygenators were supplied with an oxygen/CO_2_ mixture, the proportion of CO_2_ being varied according to arterial pH and pCO_2_ (Figure 1). In the last 6 cases, air (21% O_2_) replaced pure O_2_ as the principle gas supplied to the oxygenators with an intention of achieving an hepatic venous oxygen saturation between 55% and 75%. The gas flow was divided between both portal and arterial oxygenators by a Y-connector. Arterial pO_2_ was around 20 kPa (153 mm Hg), with 98% to 99% oxygen saturations and 65% to 85% saturation of portal blood, the difference being explained by the greater flow rate of perfusate across the portal oxygenator than arterial oxygenator, hence less gas uptake. Supplementary oxygen was required in 3 cases at the start of NESLiP. Bicarbonate was given if the perfusate pH is less than 7.2.

Normothermic ex situ liver perfusion was commenced at 20°C, and the circuit warmed to 37°C over 20 to 30 minutes; As the perfusion temperature increased, the HA and PV pressures were increased from 30 and 4 to 60 and 9 mm Hg, respectively. Perfusate gas estimations were performed every 20 to 30 minutes; hemoglobin, potassium, sodium, glucose, and lactate concentrations were also measured. Samples were initially only taken from the arterial inflow, but in the last 6 perfusions, hepatic vein gas estimations were also done. Arterial and PV flows were monitored throughout, as was bile production. Perfusate cultures were taken from each case and were negative.

### Viability

Viability was judged by assessing changes in lactate, glucose, and transaminase concentrations as well as on the ability of the liver to maintain pH without supplemental bicarbonate.

### Definitions

Postreperfusion syndrome was defined as a fall in mean arterial pressure (MAP) within 5 minutes of reperfusion in the recipient to less than 70% of the baseline value in the last 5 minutes of the anhepatic period.^8^ In the absence of an accepted definition,^9-11^ we defined vasoplegia as a fall in MAP on reperfusion to less than 50 mm Hg either sustained longer than 30 minutes and/or requiring greater than 0.15 μg/kg per minute norepinephrine, greater than 2 U/h argipressin, or infusion of epinephrine (ie, significant hypotension resistant to pressors).

### Evidence of Damage From Reactive Oxygen Species (ROS)

To seek evidence that the perfusion technique used in the first 6 livers was associated with damage from reactive oxygen species, 10 livers not used for transplantation were examined. Five of the livers had previously been perfused at high arterial pO_2_, and 5 were perfused to evaluate low oxygen tensions using air; this was used for the subsequent 6 transplants. Estimations of liver protein carbonyl concentrations and perfusate syndecan concentrations were measured as markers of ROS damage. The methods and results are in the **SDC,** http://links.lww.com/TP/B394.

### Institutional Review

Perfusion of discarded livers was approved by a research ethics committee, and the transplantation of perfused livers was approved by our institution’s New Interventional Procedures Committee. All patients gave informed consent. Where appropriate, the recipients separately consented to receive higher risk organs, such as DCD livers, according to our standard practice.

### Contemporaneous Cohort for Comparison

To provide comparative data, a contemporaneous cohort of 24 liver recipients were identified including all other fast-track recipients in the study period along with livers transplanted immediately before and after each NESLiP case of similar type (DBD/DCD).

## RESULTS

### Livers

Twelve livers underwent NESLiP over a 15-month period. Nine had been turned down by other centers, including 3 that were fast-track offers; 2 had been declined by all UK centers and were offered for research (cases 9 and 10), and 1 had a prolonged ischemic time due to the necessity to change recipient at short notice (case 12). Table 1 details the donor livers; 9 were from DCD and 3 from DBD donors, with a median age of 56 years (range, 24-67). Taking cold ischemic time to end at commencement of normothermic perfusion, the median Donor Risk Index was 2.15 (range, 1.47-3.14).^12,13^

**TABLE 1:**
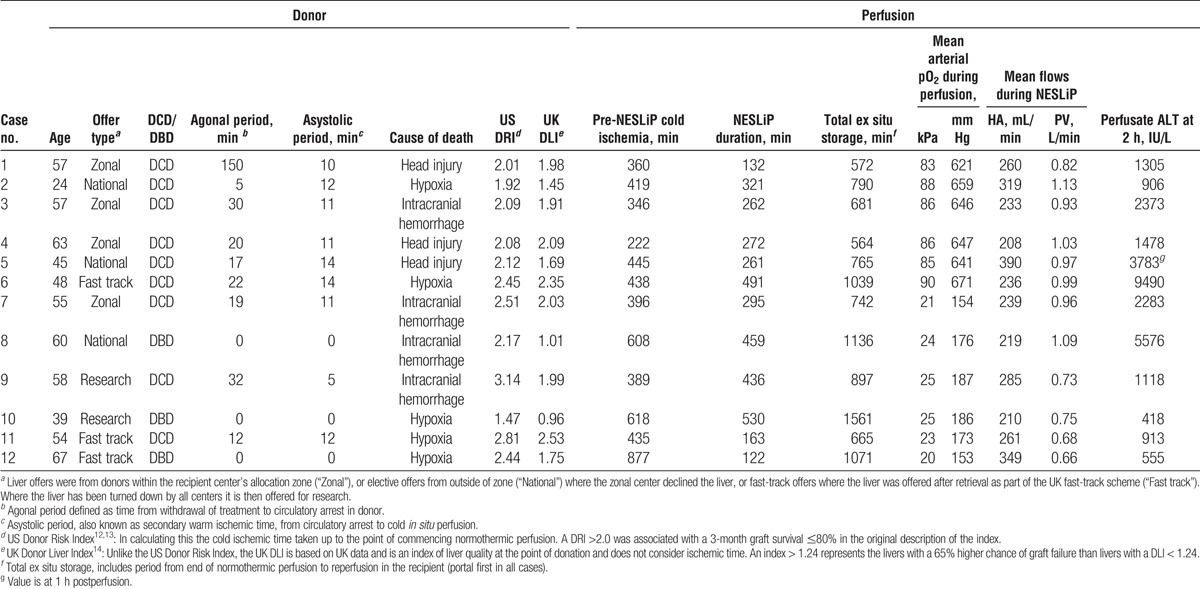
Donor and perfusion details

### Perfusions

**FIGURE 2:**
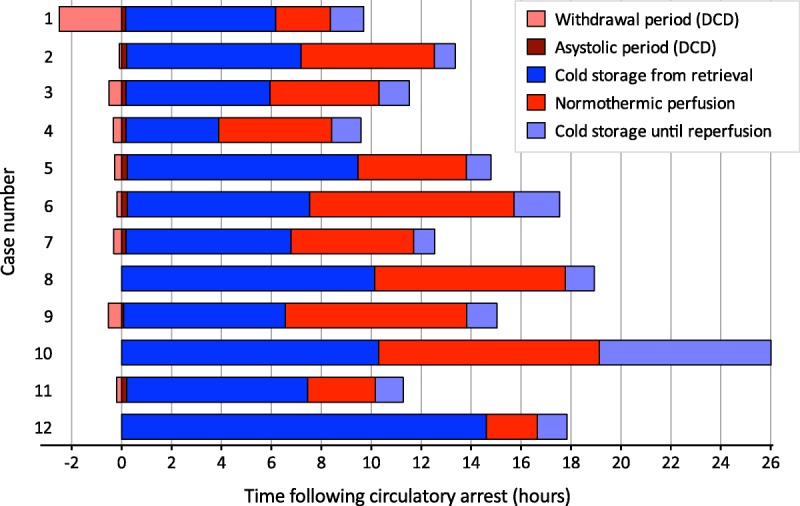
Storage times of the 12 livers, broken down by periods of storage. Horizontal bars represent individual livers, showing the periods from withdrawal of treatment, asystole, cold storage, and normothermic perfusion, removal from the machine and being cold flushed during implantation, up to reperfusion in the recipient.

Normothermic ex situ liver perfusion began after a median cold storage period of 427 minutes (range, 222-877), and livers underwent normothermic perfusion for a median of 284 minutes (range, 122-530) before being cold flushed for implantation (Figure 2). The median total time from circulatory arrest in the donor to reperfusion in the recipient was 778 minutes (12 hours, 58 minutes), with a range from 564 to 1561 minutes (9 hours, 24 minutes to 26 hours, 1 minute).

**FIGURE 3:**
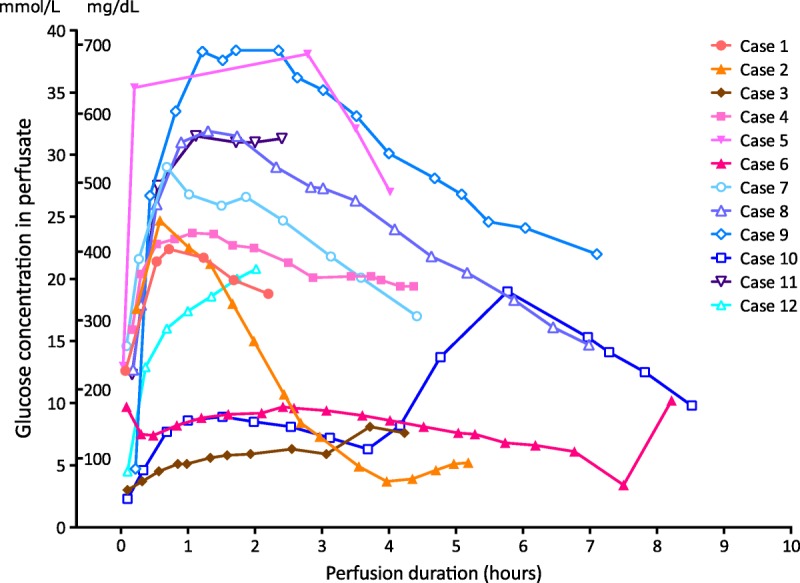
Perfusate glucose concentration during normothermic ex situ liver perfusions. Individual lines represent the change in perfusate glucose for each liver in the series; blue lines with open symbols represent livers perfused with low oxygen tensions. With 3 exceptions, there was a release of glucose on reperfusion of the liver followed by a slow fall towards normal. Note the glucose in livers 3, 6, and 10 were in the “normal” range initially. Case 10 received an infusion of glucose between 4 and 6 hours, after which there was a spontaneous and rapid fall.

**FIGURE 4:**
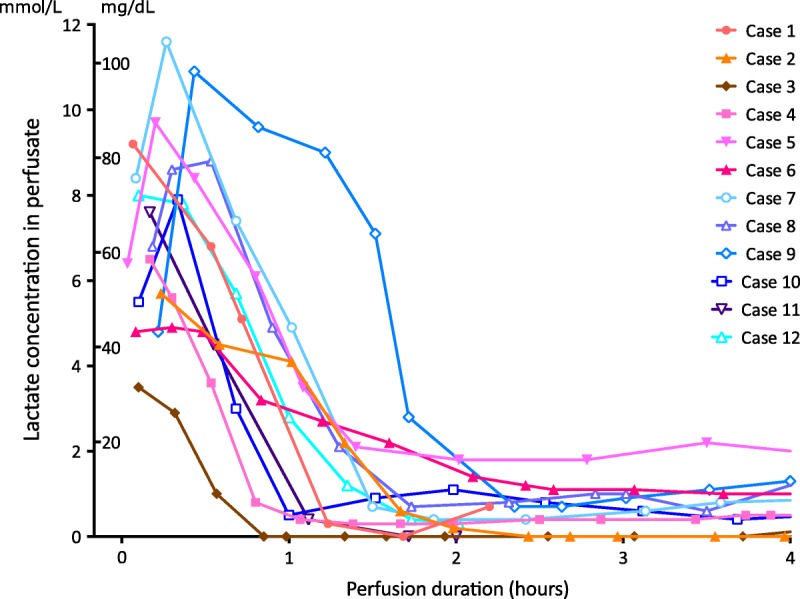
Perfusate lactate concentration during normothermic ex situ liver perfusion. Lactate concentrations for each liver in the series. Note the spontaneous fall in all cases. The livers in the last 6 cases were flushed with compound sodium lactate (Hartmann solution) before perfusion, washing out potassium and loading the liver with lactate to enable more ready assessment of a fall. Cases 5 and 9 were livers that had suffered a degree of parenchymal trauma; the delayed lactate fall in 9 and incomplete fall in 5 were interpreted in that light, with presumed ongoing lactate production in the damaged segments. Case 6, the slowest fall, suffered primary non function.

In the first 6 cases, the mean arterial pO_2_ throughout NESLiP was between 83 and 90 kPa (621-671 mm Hg), whereas for the subsequent 6 cases, oxygenation was reduced and the mean arterial pO_2_ varied between 20 and 25 kPa (153-187 mm Hg). Changes in perfusate lactate and glucose concentrations are shown in Figures 3 and 4, respectively. Livers cleared lactate at varying rates; case 6 had the slowest rate of fall of lactate, whereas case 9, a liver with a traumatic right lobe laceration and hematoma, had the most delayed fall. Case 5, a liver with trauma to the right lobe, had a brisk initial fall in lactate, but thereafter, it remained slightly raised throughout perfusion (around 2 mmol/L, 20 mg/dL).

**FIGURE 5:**
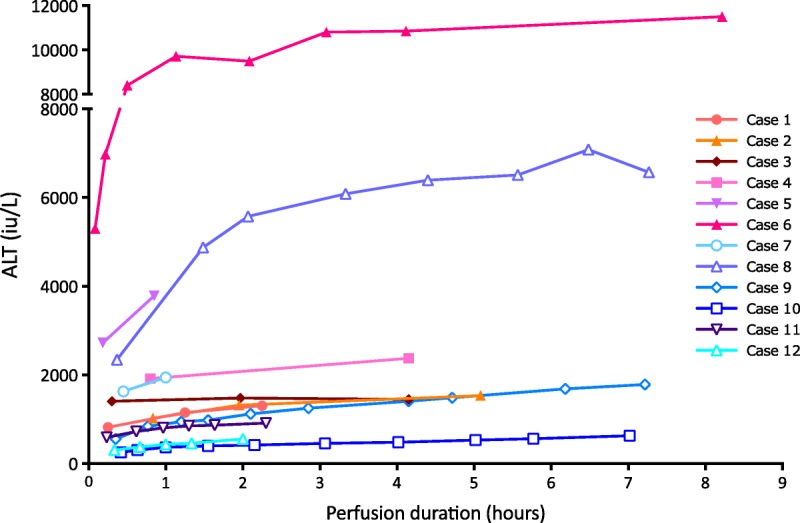
Perfusate ALT during normothermic ex situ liver perfusion. Perfusate ALTs for each liver during perfusion. Case 6 suffered primary nonfunction, and case 8 was a steatotic liver.

**FIGURE 6:**
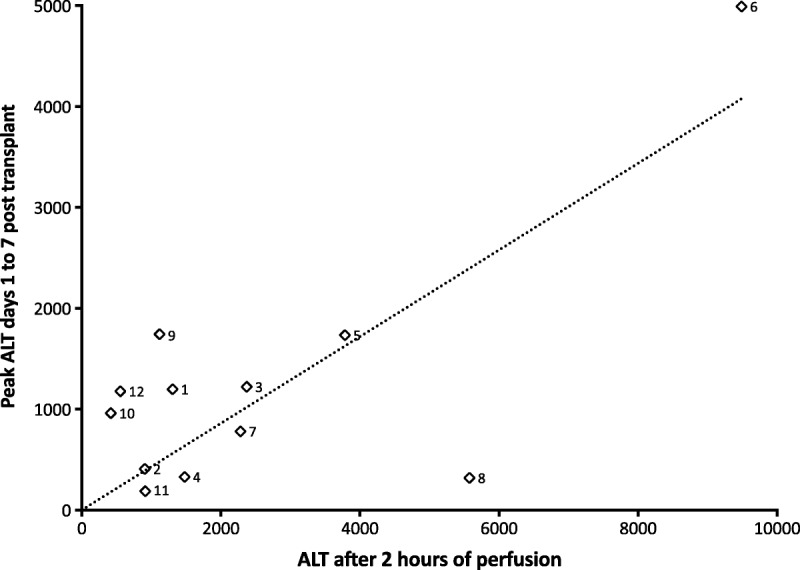
Relationship between perfusate ALT after 2 hours and the peak ALT posttransplantation. The peak ALT in the first 7 days posttransplant is plotted against the perfusate ALT after 2 hours. Note case 6 developed primary nonfunction. There was a significant correlation between the values (correlation coefficient *R*^2^ = 0.56, *p* = 0.005). The dotted line is a linear regression plot constrained through the origin.

Measurement of alanine transaminase (ALT) was performed at 1 and 2 hours after start of NESLiP, and posttransplant perfusate analysis provided additional measurements (Figure 5). The relationship between perfusate ALT concentration at 2 hours and peak ALT in the first 7 days posttransplant is shown in Figure 6.

**FIGURE 7:**
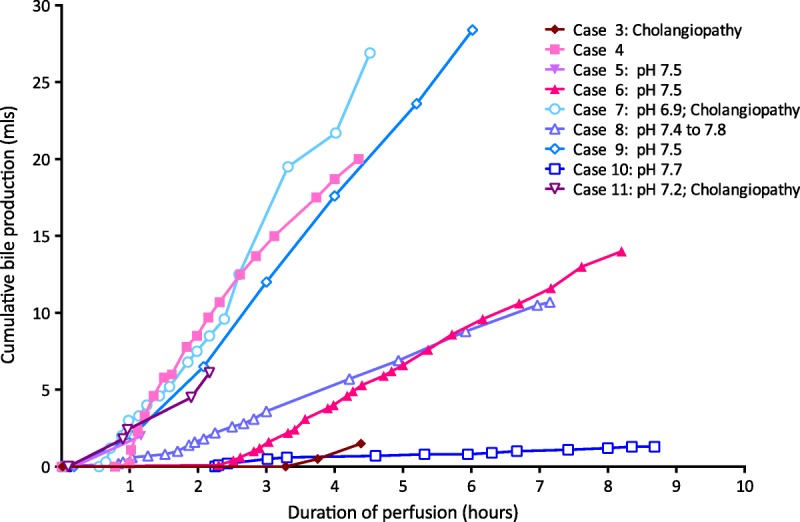
Cumulative bile production during normothermic *ex* situ liver perfusion. Bile production varied, and did not predict cholangiopathy or viability. Note that cases 7 and 11 have evidence of ischemic cholangiopathy on MRCP even though they had some of the highest rates of bile production; they also had the least alkali bile. Bile production could not be recorded in 3 livers due to occlusion of the biliary catheter. Bile salts were not added to the perfusate.

There were 2 technical problems during perfusions. One related to occlusion of the biliary catheter in 3 cases, preventing assessment of bile production (but without long term biliary sequelae); bile production in the remaining 9 cases is shown in Figure 7. The second related to occlusion of the hepatic vein catheter shortly after beginning the perfusion in case 11; it was not used in the first 6 cases.

### Recipients

The median age of recipients was 57 years (range, 46-65), with a median Model for End-Stage Liver Disease (MELD) score of 17 (range, 10-26) and a median United Kingdom End-stage Liver Disease (UKELD) score of 55 (range, 49-64) (Table 2).^15,16^ A UKELD greater than 49 corresponds to a better survival posttransplant than remaining on the waiting list. The first recipient had an uneventful course. Case 2 became hemodynamically unstable secondary to pulmonary thromboemboli during the explant, and continued to have further emboli in the postoperative period.

**TABLE 2:**
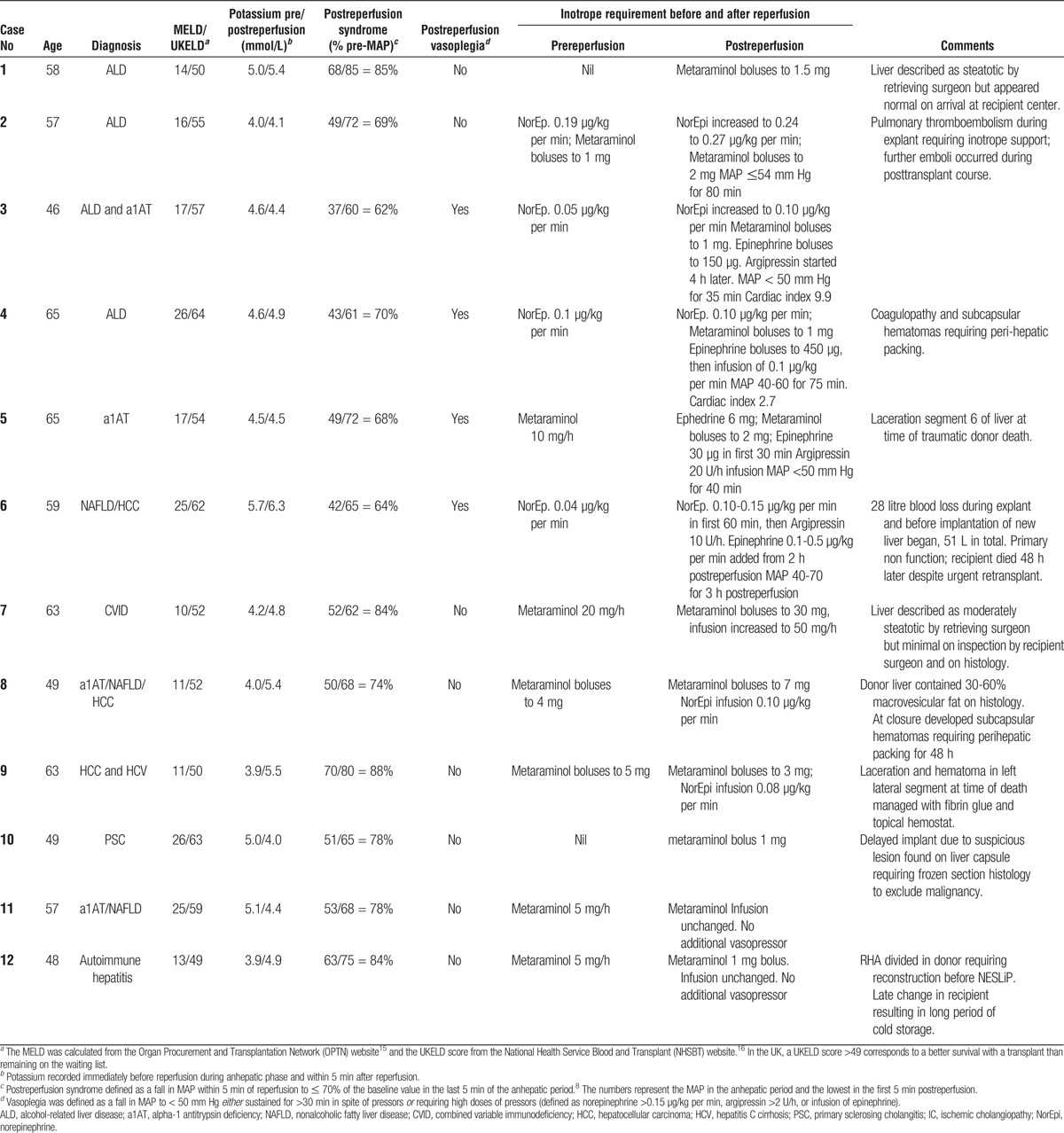
Recipient details

Case 6 had an unexpectedly difficult hepatectomy complicated by coagulopathy and severe hemorrhage, with a 28-L blood loss before implantation. The liver suffered primary nonfunction, and the patient died despite urgent retransplantation. Histology of the donor liver after explant showed extensive necrosis, but it could not be determined whether this preexisted at the time of implant or was a consequence of the inotropes given postimplantation.

Five of the first 6 recipients suffered from postreperfusion syndrome, and 4 developed sustained vasoplegia (see Table 2). None of the subsequent 6 liver recipients, who received livers treated with lower concentrations of oxygen, experienced postreperfusion syndrome or vasoplegia.

### Posttransplant Course

**FIGURE 8:**
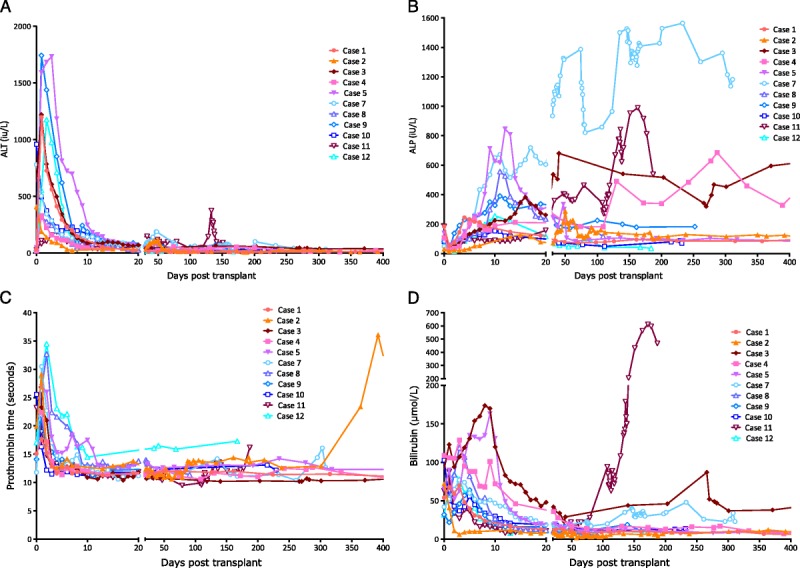
Posttransplant biochemistry. A, Posttransplant ALT (normal range, <50 IU/L). The ALT fell to normal in all patients except case 6 (not shown). The highest ALTs were in the 2 cases with parenchymal lacerations at the time of donation (cases 5 and 9). B, Posttransplant alkaline phosphatase (ALP) (normal range, <135 IU/L). Cases 3, 4, and 7 have persistently raised ALP posttransplant. Intrahepatic biliary strictures have been demonstrated by MRCP in cases 3, 7, and 11. Case 4 has a 6-cm hilar mass in conjunction with a persistently positive EBV PCR; he also had an anastomotic biliary stricture dilated 300 days posttransplant, although the relationship of this to the hilar mass is unclear. C, Posttransplant prothrombin time. The prothrombin time was persistently raised in case 12 with no obvious cause or clinical consequence. Case 2 was on warfarin for a prosthetic aortic valve, although postoperatively she was maintained initially on subcutaneous low molecular weight heparin. D, Posttransplant bilirubin. The bilirubin is raised in those cases with cholangiopathy (3, 7, and 11).

Eleven patients are alive at a median of 12 months posttransplant (range, 9-24 months). Figure 8 shows the postoperative biochemistry for the recipients. Three patients (cases 3, 7, and 11) developed cholangiopathy demonstrated on magnetic resonance cholangiopancreatography (MRCP) (Table 3). All were DCD livers, with asystolic periods of 11 to 12 minutes, agonal periods of 12 to 30 minutes, and durations from treatment withdrawal in the donor to cold in situ perfusion of 24 to 41 minutes. Case 11 had a “15- to 30-minute” cardiorespiratory arrest before admission. Cases 3 and 11 had evidence of complete destruction of periluminal and deep peribiliary glands on a postreperfusion bile duct biopsy suggesting preexisting biliary damage; case 7 did not have a bile duct biopsy. Bile collected during NESLiP in cases 7 and 11 had a pH 6.9 and 7.2, respectively; the pH of bile in case 3 was not measured.

**TABLE 3:**
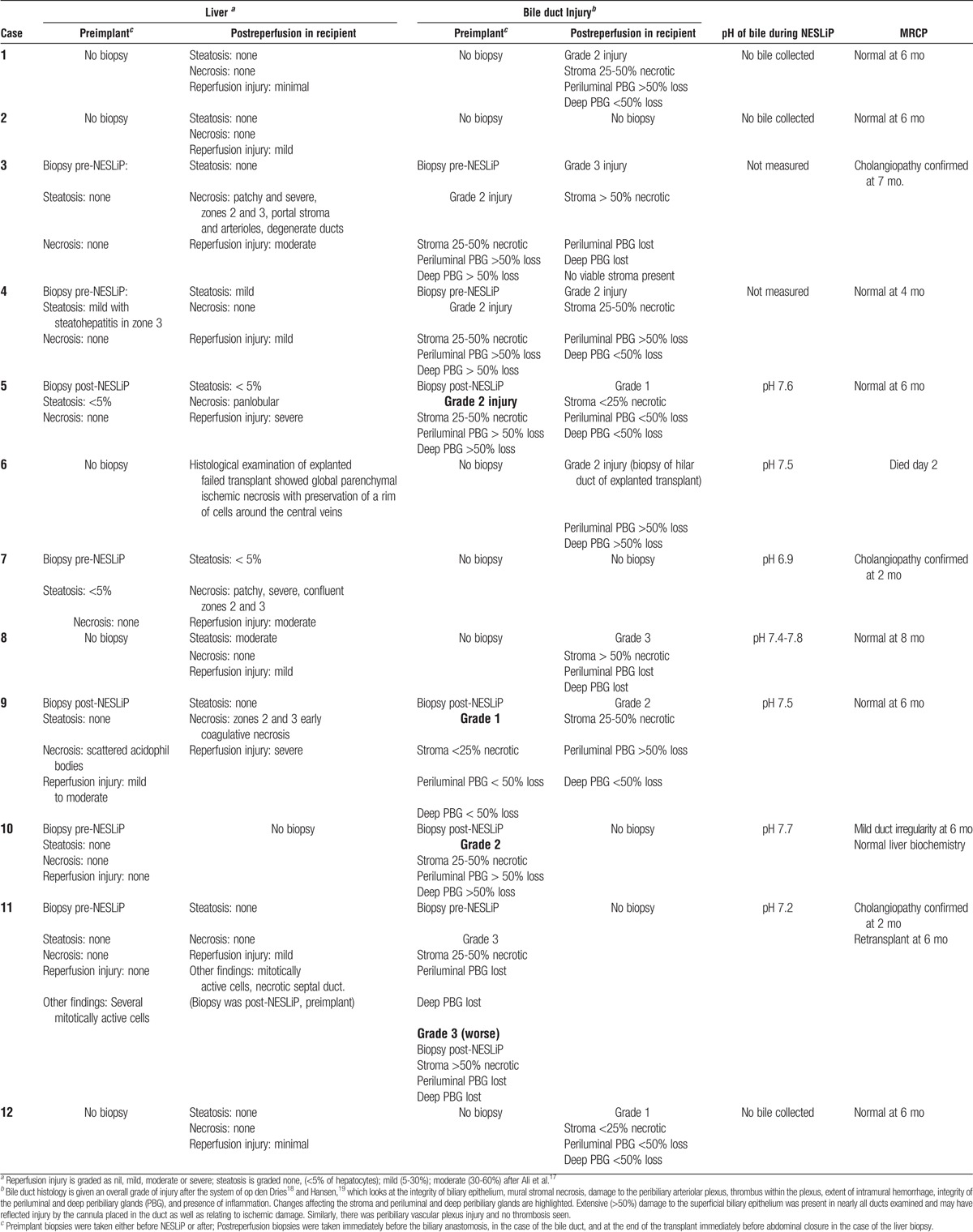
Histological findings of liver and bile duct before and after NESLiP

### Contemporaneous Cohort

Table 4 compares the outcomes of the NESLiP cases with a comparable cohort of non-NESLiP cases. The outcomes for the NESLiP cohort of declined livers are similar to those nondeclined livers not subject to NESLiP.

**TABLE 4:**
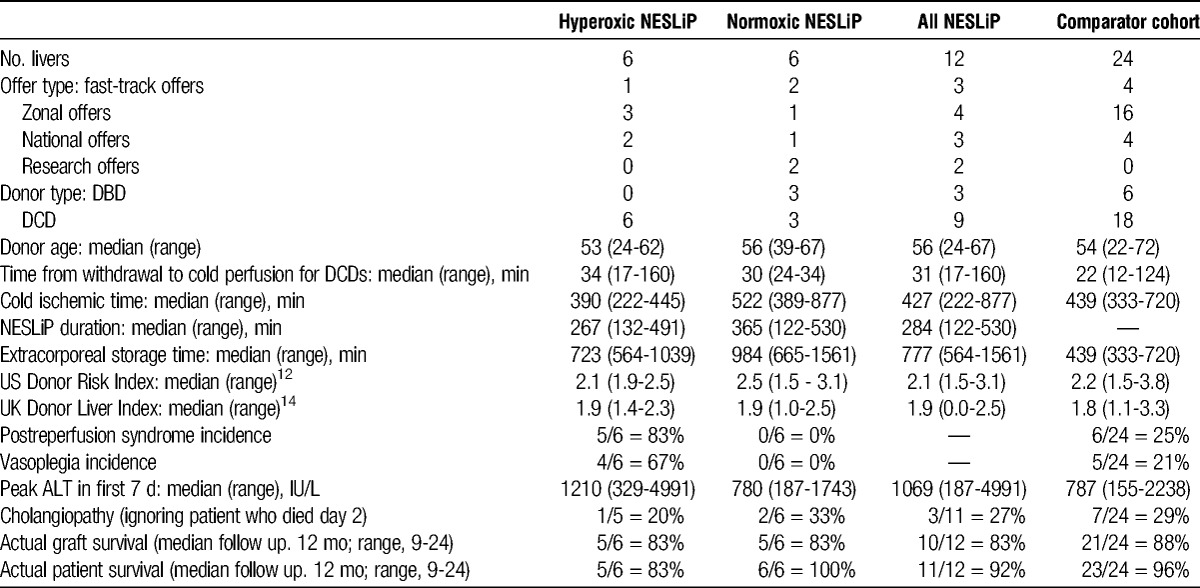
Outcomes of livers subject to NESLiP compared with a cohort of contemporaneous liver transplants

## DISCUSSION

Normothermic ex situ liver perfusion has been described in 2 settings, either used after cold storage for a period immediately before liver implantation or from the point of liver recovery from the donor until implantation.^6,7,20,21^ Here, we describe a clinical series of NESLiP after a period of cold ischemia. NESLiP was used to permit assessment of declined livers before transplantation and to stop further cold ischemic damage in marginal livers after visual inspection, for example, to assess the degree of steatosis. Using this technique we transplanted 12 livers, of which 10 had a Donor Risk Index greater than 2.0.^12^ Five livers were associated with hemodynamic instability in the recipients after reperfusion, possibly related to hyperoxia in the perfusate during NESLiP. There was no hemodynamic disturbance in recipients of the 6 livers perfused at lower oxygen tensions. Whether the high oxygen tensions were responsible for the incidence of postreperfusion syndrome, or whether the nature of the livers, or the duration of NESLiP, was responsible is not clear.

Previous investigators have described normothermic organ perfusion using high perfusate oxygen tensions. Hosgood and Nicholson^22,23^ report clinical results using a 95% O_2_ and 5% CO_2_ mixture delivered to a single oxygenator for their brief period of preimplant normothermic kidney perfusion and have not reported postreperfusion syndrome or vasoplegia. Similarly, high O_2_ partial pressures (275-650 mm Hg; 37-87 kPa) have been used in perfusion of livers in nonclinical research.^24-28^ In all the liver perfusions cited above, the liver was assessed ex vivo. In contrast, in a series of pig experiments Schön et al^29^ transplanted livers after a 4-hour period of NESLiP using a 95% O_2_/5% CO_2_ mixture to oxygenate the arterial inflow, and mixed this oxygenated blood with caval venous return to produce partially oxygenated portal blood; they reported no adverse effects posttransplant.

High concentrations of oxygen in tissues can result in formation of ROS and reactive nitrogen species (RNS), and these agents can mediate reperfusion injury and cause refractory vasoplegia, as well as damaging the endothelial glycocalyx.^30-32^ There is an extensive literature about the effects of hyperoxemia during cardiopulmonary bypass, particularly during reperfusion of the heart, where it is associated with impaired myocardial and lung perfusion after reperfusion, phenomena that are believed to be linked to ROS and RNS production.^33-35^ Similar concerns exist regarding hyperoxemia during extracorporeal membrane oxygenation and during resuscitation of neonates.^36,37^ Hyperoxia has been shown to be associated with severe hepatic reperfusion injury in a rabbit model, whereas hypoxia caused minimal reperfusion injury^38^; hypoxia before normoxic reperfusion has also been shown to prevent ROS production and depletion of antioxidants in cardiac surgery.^39^ Although we have no direct proof that hyperoxia during perfusion resulted in ROS and RNS damage to the livers we transplanted, or caused the subsequent postreperfusion syndrome and vasoplegia, we consider the higher liver protein carbonyl content and higher perfusate syndecan levels in discarded livers subject to NESLiP under hyperoxic conditions to be suggestive (**Figs. S1 and S2, SDC,** http://links.lww.com/TP/B394). Moreover, by simply reducing perfusate oxygenation, we have seen no further problems on reperfusion in the recipient.

Postreperfusion syndrome and vasoplegia have not been reported after normothermic perfusion of kidneys in the clinic. Severe liver disease is associated with impaired vasoreactivity and marked splanchnic vasodilatation,^40^ which may make the liver recipient's circulation more sensitive to inflammatory mediators associated with graft injury. In addition, the short duration of preimplant kidney perfusion may mitigate the effect. The only 1 of the first 6 livers we transplanted not to be overtly affected had the shortest exposure to NESLiP and the lowest MELD and UKELD (14 and 50, respectively). In contrast, the worst vasoplegia was seen in the recipient with a MELD of 25 and the longest exposure to NESLiP. Another factor may relate to the perfusate's hematocrit. Most of the oxygen in the perfusate is carried by red cells, with only a small proportion dissolved in solution, even at high oxygen tensions. It is possible that the low hematocrit used in normothermic kidney perfusion is protective by reducing oxygen carriage, with oxygen consumption being indicated by a very low venous oxygen saturation.

It has been suggested that the severity of reoxygenation injury in patients undergoing cardiopulmonary bypass for cyanotic heart disease, and during resuscitation of neonates, relates to depletion of endogenous antioxidants.^37,41^ Our series of declined grafts are predominantly from DCD donors and more susceptible to reperfusion injury and depletion of natural anti-oxidants, making these livers more susceptible to reoxygenation, particularly in the presence of hyperoxia. It is noteworthy that all 6 of the first cohort of livers were DCD livers, in contrast to just 3 of the second cohort, possibly contributing to the higher incidence in the first cohort.

Postreperfusion syndrome was not reported in the initial pilot study of the Metra (OrganOx, Oxford, UK) NESLiP device.^6^ In that study, the livers were placed on the machine at the point of retrieval and suffered little cold ischemia. In addition, the perfusate oxygen tension on Metra is typically between 90 and 150 mm Hg (12 and 20 kPa) (David Nasralla, personal communication). The perfusate composition used in that study was similar to the Gelofusine based perfusate we used.

We used a combination of parameters to assess the livers during perfusion. Previous hepatocellular damage was reflected in the perfusate ALT at 2 hours, by which point most enzyme washout had occurred. Lactate metabolism to glucose or glycogen occurs predominantly in periportal hepatocytes,^42^ so disturbances in lactate metabolism were considered to represent periportal hepatocyte damage or ongoing lactate production (eg, from poorly perfused parenchyma). Flushing the liver with compound sodium lactate before NESLiP provided a higher baseline lactate whose metabolism could be followed.

Glycogenolysis is an ATP-independent process that continues during cold storage and is enhanced at reperfusion^43,44^ and explains the raised perfusate glucose seen in many of the NESLiP cases. Although a raised glucose is commonly observed during NESLiP, a normal glucose might be a manifestation of glycogen exhaustion and/or extensive lobular damage, or it may signify minimal ischemia. Hence, although a raised perfusate glucose may be a marker of moderate ischemia, a normal glucose may paradoxically represent either minimal or severe ischemic damage. This was seen in case 6, where the glucose was normal, probably as a result of global lobular injury rather than implying a good liver as first thought. To rule out severe lobular injury in case 10, a glucose challenge was given after which the glucose fell rapidly. The fall in glucose observed is explained by glucose entry into the liver via the insulin-independent GLUT2 transporter, and its subsequent incorporation into glycogen (data not shown).

Hepatic regulation of acid-base balance depends upon the differential metabolism of glutamine along the lobule.^45^ An inability to regulate pH, with worsening acidosis, was considered to signify pan-lobular hepatocyte damage,^46,47^ and it is noteworthy that case 6 had the greatest tendency to acidosis during perfusion (data not shown).

Bile production has been suggested as a sensitive marker of liver viability during NESLiP.^48-50^ It is a complex process dependent on the integrity of many facets of liver function. Bile acids are secreted predominantly in zones 1 and 2, whereas bicarbonate is secreted in zone 3.^51^ Viability of the cholangiocytes will also influence the amount and quality of bile production. Figure 7 shows varying patterns of bile production. Case 10, the poorest bile producer, is a liver with satisfactory function and minimal evidence of cholangiopathy, whereas cases 7 and 11 are among the best producers of bile that have developed clinically significant cholangiopathy. Case 6, the liver suffering primary non function, also made a reasonable amount of bile. It is unclear from our series how much emphasis should be placed on the amount of bile produced, but the ability to produce an alkali bile might be a more significant marker of cholangiocyte integrity. Where bile was produced and its pH measured, only the livers not able to produce bile with a pH greater than 7.4 went on to develop significant cholangiopathy.

The high incidence of cholangiopathy in this series is in contrast to reports from researchers using cold machine perfusion, albeit of less marginal livers,^52^ but is similar to the contemporaneous non-NESLiP cohort. The presence of severe duct injury visible on duct biopsies before normothermic perfusion in cases 3 and 11 suggests this might be a donor phenomenon and not related to the perfusion technique.

In summary, our report shows that a period of normothermic perfusion before transplantation can allow biochemical assessment of liver function and arrest of cold ischemia. Hyperoxic perfusates were associated with postreperfusion vasoplegia and hemodynamic instability, possibly as a consequence of release of ROS and RNS, whereas lower perfusate oxygen tensions were associated with an uneventful reperfusion, although the numbers are too small to confidently make a causal association. We noted a high incidence of cholangiopathy which was associated with an inability to produce an alkali pH during NESLiP.

## Supplementary Material

SUPPLEMENTARY MATERIAL
