# Supplementary material for: Normothermic Perfusion in the Assessment and Preservation of Declined Livers Before Transplantation: Hyperoxia and Vasoplegia—Important Lessons From the First 12 Cases
Source: Transplantation. 2017 Jan 24;101(5):1084–98. doi: 10.1097/TP.0000000000001661 (PMC5642347; doi:10.1097/TP.0000000000001661)
Supplement: SUPPLEMENTARY MATERIAL [file tp-101-1084-s001.docx]

**Supplementary digital content**

One possible explanation of the reperfusion problems encountered in 5 recipients was damage due to reactive oxygen (ROS) and reactive nitrogen species (RNS) released by the donor liver on reperfusion. In order to investigate this further *in vitro* tests were performed on 10 livers that were not used for transplantation but were subjected to normothermic perfusion (as discussed in methods). Of the 10 discarded livers, 5 were subjected to NESLiP with high oxygen tension in the perfusate and 5 to NESLiP at more physiological oxygen tensions

***Protein carbonyl estimation***

Protein oxidation by ROS results in formation of carbonyl groups (aldehydes and ketones) on protein side chains (1). Wedge biopsies taken at 3 hours following the start of normothermic perfusion had been immediately immersed in liquid nitrogen and stored at -70°C. Protein carbonyl estimation was performed using a commercial ELISA assay (Biocell, Papatoetoe, New Zealand). Higher levels of protein carbonyls were seen in livers exposed to higher oxygen tensions (p=0.04, Mann Whitney test. Supplementary figure 1).

***Syndecan-1 (CD138)***

Syndecan-1 is part of the endothelial glycocalyx and is released as a consequence of damage to the glycocalyx by ROS and reactive nitrogen species (RNS) (2). Perfusate samples taken 3 to 4 hours after the start of perfusion were centrifuged to remove cells and the supernatant stored at -70°C. Syndecan-1 concentrations in the perfusates were estimated using a commercial enzyme linked immunoabsorbent assay (ELISA) (Abcam, Cambridge, UK), according to the manufacturer’s instructions. Syndecan concentrations were non significantly higher in the perfusates subject to higher oxygen tensions than those subject to lesser oxygenation (supplementary figure 2).

**Reference**

1. Dalle-Donne I, Rossi R, Giustarini D, Milzani A, Colombo R. Protein carbonyl groups as biomarkers of oxidative stress. Clinica Chimica Acta 2003; 329: 23-38.

2. van Golen RF, van Gulik TM, Heger M. Mechanistic overview of reactive species-induced degradation of the endothelial glycocalyx during hepatic ischemia/reperfusion injury. Free Radic Biol Med 2012;52(8):1382-1402

**Supplementary figure 1. Protein carbonyls at 3 hours**

The median carbonyl concentration in biopsies was higher in livers exposed to a hyperoxic perfusate.

**Supplementary figure 2. Syndecan assay on perfusate samples taken after 3 to 4 hours of perfusion**

The median perfusate syndecan-1 concentration was higher in livers exposed to hyperoxia, suggesting less glycocalyx damage in the normoxic group.
