# Supplementary material for: Normothermic Perfusion in the Assessment and Preservation of Declined Livers Before Transplantation: Hyperoxia and Vasoplegia—Important Lessons From the First 12 Cases
Source: Transplantation. 2017 Jan 24;101(5):1084–98. doi: 10.1097/TP.0000000000001661 (PMC5642347; doi:10.1097/TP.0000000000001661)
Supplement: SUPPLEMENTARY MATERIAL [file tp-101-1084-s002.docx]

**Normothermic perfusion in the assessment and preservation of declined livers prior to transplantation: hyperoxia and vasoplegia – important lessons from the first 12 cases.**

**Authors**

Christopher J. E. Watson^1^, MD

Vasilis Kosmoliaptsis^1^, PhD.

Lucy V. Randle^1^

Alexander E. Gimson^2^, MB BS

Rebecca Brais^3^, BA BM

John R. Klinck^4^, MD

Mazin Hamed^1^, MRCS

Anastasia Tsyben^5^ BA

Andrew J. Butler^1^, MChir

**Affiliations**

1. University of Cambridge Department of Surgery, Addenbrooke’s Hospital, Cambridge, CB2 0QQ, UK; the NIHR Cambridge Biomedical Research Centre and the NIHR Blood and Transplant Research Unit in Organ Donation and Transplantation at the University of Cambridge

2. Department of Medicine, Addenbrooke’s Hospital, Cambridge, UK

3. Department of Pathology, Addenbrooke’s Hospital, Cambridge, UK

4. Division of Perioperative Care, Addenbrooke’s Hospital, Cambridge, UK

5. University of Cambridge School of Clinical Medicine, Addenbrooke’s Hospital, Cambridge, UK

**Corresponding author:** Chris Watson. Email: cjew2@cam.ac.uk

University of Cambridge Department of Surgery, Box 202, Addenbrooke’s Hospital, Cambridge, CB2 0QQ, UK.

**Authorship page**

Contributions to work.

CJW, VK, AJB conceived of the programme; they together with LVR, AEG, RB, and JRK took part in the analysis and interpretation of the data; MH and AT undertook the *in vitro* analyses and also took part in the interpretation of the data. All authors reviewed the manuscript and approved the final manuscript, and all agree to be accountable for all aspects of the work.

Disclosures

Andrew Butler is a joint holder of a patent on the design of the perfusion circuit used by the OrganOx Metra liver perfusion device. Lucy Randle is now an employee of OrganOx. The authors of this manuscript declare no other conflicts of interest.

Funding

The background work to this series, and the *in vitro* work described here, was funded by Addenbrooke’s Charitable Trust. XVivo Perfusion (Göteburg, Sweden) subsidized the cost of the Steen Solution used in 3 of these cases.

**Abbreviations**

a1AT alpha-1 anti-trypsin deficiency

ALD Alcohol related liver disease

ALP Alkaline phosphatase

ALT Alanine transaminase

AST Aspartate transaminase

BMI Body mass index

CVID Combined variable immunodeficiency

DBD Donation after brain death

DCD Donation after circulatory death

DRI Donor risk index

HCC Hepatocellular carcinoma

HCV Hepatitis C cirrhosis

IC Ischemic cholangiopathy

LDI United Kingdom Liver Donor Index

MAP mean arterial pressure

MELD Model for end-stage liver disease score (MELD).

MRCP Magnetic resonance cholangiopancreatography

NAFLD Nonalcoholic fatty liver disease

NESLiP Normothermic *ex situ* liver perfusion

NorEpi Norepinephrine (noradrenaline)

pCO_2_ Partial pressure of carbon dioxide in the perfusate

pO_2_ Partial pressure of oxygen in the perfusate

PSC Primary sclerosing cholangitis

UK United Kingdom

USA United States of America

**Abstract**

*Background*

A programme of normothermic *ex situ* liver perfusion (NESLiP) was developed to facilitate better assessment and use of marginal livers, while minimising cold ischaemia.

*Methods*

Declined marginal livers and those offered for research were evaluated. NESLiP was performed using an erythrocyte–based perfusate. Viability was assessed with reference to biochemical changes in the perfusate.

*Results*

12 livers (9 from circulatory death (DCD) and 3 from brain-dead donors), median Donor Risk Index 2.15, were subjected to NESLiP for a median 284 minutes (range 122-530) after an initial cold storage period of 427 minutes (range 222-877). The first 6 livers were perfused at high perfusate oxygen tensions, and the subsequent 6 at near-physiologic oxygen tensions. After transplantation, 5 of the first 6 recipients developed postreperfusion syndrome and 4 had sustained vasoplegia; 1 recipient experienced primary nonfunction in conjunction with a difficult explant. The subsequent 6 liver transplants, with livers perfused at lower oxygen tensions, reperfused uneventfully. Three DCD liver recipients developed cholangiopathy, and this was associated with an inability to produce an alkali bile during NESLiP.

*Conclusions*

NESLiP enabled assessment and transplantation of 12 livers that may otherwise not have been used. Avoidance of hyperoxia during perfusion may prevent postreperfusion syndrome and vasoplegia, and monitoring biliary pH, rather than absolute bile production, may be important in determining the likelihood of posttransplant cholangiopathy. NESLiP has the potential to increase liver utilization, but more work is required to define factors predicting good outcomes.

**Introduction**

The last decade has seen increasing numbers of patients being listed for liver transplantation but a decline in the overall quality of available donor organs, with larger numbers of older donors, donors dying from hypoxic brain injury, and following circulatory death (DCD) ^1^. As a consequence in the US in 2014, 10% of livers recovered from deceased donors were not transplanted in spite of over 3000 potential recipients dying or being removed from the waiting list in the same year ^1^. The situation is similar in the UK, where 19% of patients either die or are removed from the waiting list within 2 years of listing, while 8% of livers recovered from donation after brain death (DBD) donors, and 26% of livers recovered from DCD donors, were not transplanted ^2,3^.

The principle reason for discard of a liver that has been recovered for transplantation is fear that the liver will not provide life sustaining function after transplantation, usually in the setting of steatosis, prolonged warm ischaemia, adverse haemodynamic characteristics during the DCD withdrawal phase, or prolonged cold ischaemia ^4^. Although the liver may have functioned well in the donor, warm and cold ischaemia impose an unpredictable injury on the liver that may manifest only following reperfusion in the recipient.

In an effort to increase the utilisation of livers, the UK introduced a “fast-track liver offer scheme” in 1997 to place livers “that have been declined for any reason, or have yet to be accepted, at or after cross-clamp” in the donor ^5^. A simultaneous offer is made to every UK centre that has not previously declined the liver. Such livers are typically reported to be abnormal (eg steatotic, poor in situ perfusion), or were associated with a long withdrawal phase. Since fast track offering usually takes place after organ recovery, such livers are usually associated with long cold ischaemic times. Livers not accepted on the fast-track scheme may be allocated for research if there is appropriate consent. In the period of this report, 587 livers were fast-tracked, with 11% being transplanted.

The ability to undertake normothermic *ex situ* machine perfusion of livers (NESLiP) has introduced a new dimension to the assessment of donor livers before implantation, and enables cold ischaemia to be halted while a recipient is prepared for transplantation. While an initial clinical study has shown encouraging results from normothermic preservation throughout the period of extra-corporeal storage ^6^, there are few reports of the use of NESLiP in the assessment of marginal liver grafts ^7^.

Against the background of increasingly marginal liver offers we established a clinical programme of NESLiP in our centre, targeting livers that were considered potentially viable, but where the ischaemic time would be unreasonably long or there was uncertainty about the liver based on the subjective opinion of the retrieving surgeon. The programme was paused after 6 cases due to adverse events, and following further investigation and subsequent reduction in the level of oxygenation during perfusion, was restarted. This paper describes our initial experience of 12 cases, and the lessons learned that we believe will benefit all investigators in this area.

**Materials and Methods**

*Livers and Recipients*

Routine national and zonal liver offers, as well as those offered through the UK fast-track liver offering scheme were considered. Livers that had been offered directly for research were also considered. NESLiP was typically considered where there was uncertainty about the liver such that the recipient hepatectomy could not start before visualising the liver, and the prolongation of cold ischaemic time that this imposed would have deleterious consequences on the liver. Livers were offered to the highest priority patient (by biochemical or clinical criteria) of suitable size and blood group match who had previously consented for a liver of that type and separately consented for it to undergo NESLiP.

*Preparation*

Livers were prepared for transplantation in a standard manner. In addition, a 6Fr infant feeding tube was placed into the bile duct to drain bile and the cystic duct was ligated in continuity. Infusion cannulae were tied into the portal vein (PV) and celiac trunk (HA). In the last 6 livers a 6Fr catheter was also sutured with its tip in the right hepatic vein for direct sampling of venous effluent. The liver was flushed with either a litre of succinylated gelatin (Gelofusine, BBraun Medical, UK) (cases 1 to 6) or with compound sodium lactate (Hartmann’s solution, Baxter, UK) (cases 7 to 12) at room temperature prior to NESLiP (figure 1). Flushing was performed to remove residual UW solution, in particular its potassium. Hartmann’s solution was used in the last 6 cases to provide lactate as a substrate to enhance monitoring of its clearance during perfusion (1 litre contains 29mmol lactate).

Just prior to explanting the recipient’s diseased liver, NESLiP was stopped and the donor liver flushed with 2 litres ice-cold UW solution (Belzer-UW, Bridge to Life, London, UK). Implantation involved a caval-preserving cavo-cavostomy anastomosis. The liver was flushed with a litre of succinylated gelatin at room temperature before reperfusion via the portal vein; arterial reperfusion followed portal reperfusion.

*Perfusion method*

NESLiP was performed using a Liver Assist device (Organ Assist, Groningen, the Netherlands), which provides pressure-regulated flow from 2 independent pumps and oxygenators supplying portal vein and hepatic artery respectively. The perfusate comprised 3 units of leucocyte-depleted washed red cells which had a variable volume of around 1 litre. The red cells were added to a litre of either succinylated gelatin or Steen solution (Xvivo Perfusion, Göteborg, Sweden) (cases 6 to 8 only) and supplemented with 30mmol sodium bicarbonate, 25000units (50mg) heparin, antibiotics, calcium chloride, magnesium sulphate, and amino acids (Aminoven-25, Fresenius Kabi Ltd, UK). The final haemoglobin concentration was a median 6.1g/dL (range 5.1 to 7.4), hematocrit 0.18 (range 0.16 to 0.22). Epoprostenol 2µg/h was given by infusion, and insulin was given either as an infusion or bolus. Bile salts were not administered.

In the first 6 perfusions the oxygenators were supplied with an oxygen/ CO_2_ mixture, the proportion of CO_2_ being varied according to arterial pH and pCO_2_ (figure 1). In the last 6 cases air (21%O_2_) replaced pure O_2_ as the principle gas supplied to the oxygenators with an intention of achieving an hepatic venous oxygen saturation between 55 and 75%. The gas flow was divided between both portal and arterial oxygenators by a Y-connector. Arterial pO_2_ was around 20kPa (153mmHg), with 98-99% oxygen saturations and 65-85% saturation of portal blood, the difference being explained by the greater flow rate of perfusate across the portal oxygenator than arterial oxygenator, hence less gas uptake. Supplementary oxygen was required in 3 cases at the start of NESLiP. Bicarbonate was given if the perfusate pH <7.2.

NESLiP was commenced at 20°C and the circuit warmed to 37°C over 20-30 minutes; As the perfusion temperature increased the HA and PV pressures were increased from 30mmHg and 4mmHg, to 60mmHg and 9mmHg respectively. Perfusate gas estimations were performed every 20 – 30 minutes; haemoglobin, potassium, sodium, glucose, and lactate concentrations were also measured. Samples were initially only taken from the arterial inflow, but in the last 6 perfusions hepatic vein gas estimations were also done. Arterial and portal vein flows were monitored throughout, as was bile production. Perfusate cultures were taken from each case and were negative.

*Viability*

Viability was judged by assessing changes in lactate, glucose, and transaminase concentrations as well as on the ability of the liver to maintain pH without supplemental bicarbonate.

*Definitions*

Post reperfusion syndrome was defined as a fall in mean arterial pressure (MAP) within 5 minutes of reperfusion in the recipient to less than 70% of the baseline value in the last 5 minutes of the anhepatic period ^8^. In the absence of an accepted definition ^9-11^, we defined vasoplegia as a fall in MAP on reperfusion to <50mmHg either sustained for >30 minutes and/or requiring >0.15µg/kg/min norepinephrine, >2u/h argipressin, or infusion of epinephrine (ie significant hypotension resistant to pressors).

*Evidence of damage from reactive oxygen species (ROS)*

To seek evidence that the perfusion technique used in the first 6 livers was associated with damage from reactive oxygen species, 10 livers not used for transplantation were examined. Five of the livers had previously been perfused at high arterial pO_2_, and 5 were perfused to evaluate low oxygen tensions using air; this was used for the subsequent 6 transplants. Estimations of liver protein carbonyl concentrations and perfusate syndecan concentrations were measured as markers of ROS damage. The methods and results are in the SDC, http://links.lww.com/TP/B394.

*Institutional Review*

Perfusion of discarded livers was approved by a Research Ethics Committee, and the transplantation of perfused livers was approved by our institution’s New Interventional Procedures Committee. All patients gave informed consent. Where appropriate, the recipients separately consented to receive higher risk organs, such as DCD livers, according to our standard practice.

*Contemporaneous cohort for comparison*

In order to provide comparative data, a contemporaneous cohort of 24 liver recipients were identified including all other fast track recipients in the study period along with livers transplanted immediately before and after each NESLiP case of similar type (DBD/DCD).

**Results**

Livers

12 livers underwent NESLiP over a 15 month period. Nine had been turned down by other centres, including 3 that were fast-track offers; 2 had been declined by all UK centres and were offered for research (cases 9 and 10), and one had a prolonged ischaemic time due to the necessity to change recipient at short notice (case 12). Table 1 details the donor livers; 9 were from DCD and 3 from DBD donors, with a median age of 56 years (range 24 to 67). Taking cold ischaemic time to end at commencement of normothermic perfusion, the median Donor Risk Index was 2.15 (range 1.47 to 3.14) ^12,13^.

Perfusions

NESLiP began after a median cold storage period of 427 minutes (range 222 to 877), and livers underwent normothermic perfusion for a median of 284 minutes (range 122 to 530) before being cold flushed for implantation (figure 2). The median total time from circulatory arrest in the donor to reperfusion in the recipient was 778 mins (12h 58min), with a range from 564 to 1561 minutes (9h 24min to 26h 1min).

In the first 6 cases the mean arterial pO_2_ throughout NESLiP was between 83 and 90kPa (621 - 671mmHg), whereas for the subsequent 6 cases oxygenation was reduced and the mean arterial pO_2_ varied between 20 and 25kPa (153 – 187mmHg). Changes in perfusate lactate and glucose concentrations are shown in figures 3 and 4 respectively. Livers cleared lactate at varying rates; case 6 had the slowest rate of fall of lactate while case 9, a liver with a traumatic right lobe laceration and haematoma, had the most delayed fall. Case 5, a liver with trauma to the right lobe, had a brisk initial fall in lactate but thereafter it remained slightly raised throughout perfusion (around 2mmol/L, 20mg/dL).

Measurement of ALT was performed at one and 2 hours after start of NESLiP, and posttransplant perfusate analysis provided additional measurements (figure 5). The relationship between perfusate ALT concentration at 2 hours and peak ALT in the first 7 days posttransplant is shown in figure 6.

There were 2 technical problems during perfusions. One related to occlusion of the biliary catheter in 3 cases, preventing assessment of bile production (but without long term biliary sequelae); bile production in the remaining 9 cases is shown in figure 7. The second related to occlusion of the hepatic vein catheter shortly after beginning the perfusion in case 11; it was not used in the first 6 cases.

Recipients (table 2)

The median age of recipients was 57 years (range 46 to 65), with a median model for end-stage liver disease (MELD) score of 17 (range 10 to 26) and a median United Kingdom End-stage Liver Disease (UKELD) score of 55 (range 49 to 64) ^14,15^. A UKELD>49 corresponds to a better survival posttransplant than remaining on the waiting list. The first recipient had an uneventful course. Case 2 became haemodynamically unstable secondary to pulmonary thromboemboli during the explant, and continued to have further emboli in the postoperative period.

Case 6 had an unexpectedly difficult hepatectomy complicated by coagulopathy and severe haemorrhage, with a 28L blood befor implantation. The liver suffered primary nonfunction and the patient died in spite of urgent retransplantation. Histology of the donor liver after explant showed extensive necrosis, but it could not be determined whether this preexisted at the time of implant or was a consequence of the inotropes given post implantation.

Five of the first 6 recipients suffered from post reperfusion syndrome, and 4 developed sustained vasoplegia (see table 2). None of the subsequent 6 liver recipients, who received livers treated with lower concentrations of oxygen, experienced post reperfusion syndrome or vasoplegia.

*Posttransplant course*

Eleven patients are alive at a median 12 months posttransplant (range 9 to 24 months). Figure 8 shows the postoperative biochemistry for the recipients. Three patients (cases 3, 7, and 11) developed cholangiopathy demonstrated on magnetic resonance cholangiopancreatography (MRCP) (table 3). All were DCD livers, with asystolic periods of 11 to 12 minutes, agonal periods of 12 to 30 minutes, and durations from treatment withdrawal in the donor to cold in situ perfusion of 24 to 41 minutes. Case 11 had a “15 to 30 minute” cardiorespiratory arrest prior to admission. Cases 3 and 11 had evidence of complete destruction of periluminal and deep peribiliary glands on a postreperfusion bile duct biopsy suggesting preexisting biliary damage; case 7 did not have a bile duct biopsy. Bile collected during NESLiP in cases 7 and 11 had a pH 6.9 and 7.2 respectively; the pH of bile in case 3 was not measured.

*Contemporaneous cohort*

Table 4 compares the outcomes of the NESLiP cases with a comparable cohort of non-NESLiP cases. The outcomes for the NESLiP cohort of declined livers are similar to those nondeclined livers not subject to NESLiP.

**Discussion.**

Normothermic *ex situ* liver perfusion (NESLiP) has been described in 2 settings, either used following cold storage for a period immediately before liver implantation or from the point of liver recovery from the donor until implantation ^6,7,16,17^. Here, we describe a clinical series of NESLiP following a period of cold ischaemia. NESLiP was used to permit assessment of declined livers prior to transplantation and to stop further cold ischemic damage in marginal livers following visual inspection, for example, to assess the degree of steatosis. Using this technique we transplanted 12 livers, of which 10 had a DRI>2.0^12^. Five livers were associated with haemodynamic instability in the recipients following reperfusion, possibly related to hyperoxia in the perfusate during NESLiP. There was no haemodynamic disturbance in recipients of the 6 livers perfused at lower oxygen tensions. Whether the high oxygen tensions were responsible for the incidence of post reperfusion syndrome, or whether the nature of the livers, or the duration of NESLiP, was responsible is not clear.

Previous investigators have described normothermic organ perfusion using high perfusate oxygen tensions. Hosgood and Nicholson report clinical results using a 95% O_2_ and 5% CO_2_ mixture delivered to a single oxygenator for their brief period of preimplant normothermic kidney perfusion, and have not reported postreperfusion syndrome or vasoplegia ^18,19^. Similarly, high O_2_ partial pressures (275-650mmHg, 37 to 87kPa) have been used in perfusion of livers in nonclinical research ^20-24^. In all the liver perfusions cited above the liver was assessed *ex vivo*. In contrast, in a series of pig experiments Schön *et al* ^25^ transplanted livers following a 4 hour period of NESLiP using a 95%O_2_/5%CO_2_ mixture to oxygenate the arterial inflow, and mixed this oxygenated blood with caval venous return to produce partially oxygenated portal blood; they reported no adverse effects posttransplant.

High concentrations of oxygen in tissues can result in formation of reactive oxygen and nitrogen species (ROS and RNS), and these agents can mediate reperfusion injury and cause refractory vasoplegia, as well as damaging the endothelial glycocalyx ^26-28^. There is an extensive literature about the effects of hyperoxaemia during cardiopulmonary bypass, particularly during reperfusion of the heart, where it is associated with impaired myocardial and lung perfusion following reperfusion, phenomena that are believed to be linked to ROS and RNS production ^29-31^. Similar concerns exist regarding hypoxaemia during extracorporeal membrane oxygenation and during resuscitation of neonates ^32,33^. Hyperoxia has been shown to be associated with severe hepatic reperfusion injury in a rabbit model, whereas hypoxia caused minimal reperfusion injury ^34^; hypoxia before normoxic reperfusion has also been shown to prevent ROS production and depletion of antioxidants in cardiac surgery^35^. Although we have no direct proof that hyperoxia during perfusion resulted in ROS and RNS damage to the livers we transplanted, or caused the subsequent post reperfusion syndrome and vasoplegia, we consider the higher liver protein carbonyl content and higher perfusate syndecan levels in discarded livers subject to NESLiP under hyperoxic conditions to be suggestive (Figs. S1 and S2, SDC, http://links.lww.com/TP/B394). Moreover by simply reducing perfusate oxygenation we have seen no further problems on reperfusion in the recipient.

Post reperfusion syndrome and vasoplegia have not been reported following normothermic perfusion of kidneys in the clinic. Severe liver disease is associated with impaired vasoreactivity and marked splanchnic vasodilatation ^36^, which may make the liver recipient’s circulation more sensitive to inflammatory mediators associated with graft injury. In addition, the short duration of preimplant kidney perfusion may mitigate the effect. The only one of the first 6 livers we transplanted not to be overtly affected had the shortest exposure to NESLiP and the lowest MELD and UKELD (14 and 50 respectively). In contrast the worst vasoplegia was seen in the recipient with a MELD of 25 and the longest exposure to NESLiP. Another factor may relate to the perfusate’s haematocrit. Most of the oxygen in the perfusate is carried by red cells, with only a small proportion dissolved in solution, even at high oxygen tensions. It is possible that the low haematocrit used in normothermic kidney perfusion is protective by reducing oxygen carriage, with oxygen consumption being indicated by a very low venous oxygen saturation.

It has been suggested that the severity of reoxygenation injury in patients undergoing cardiopulmonary bypass for cyanotic heart disease, and during resuscitation of neonates, relates to depletion of endogenous antioxidants ^33,37^. Our series of declined grafts are predominantly from DCD donors and more susceptible to reperfusion injury and depletion of natural anti-oxidants, making these livers more susceptible to reoxygenation, particularly in the presence of hyperoxia. It is noteworthy that all 6 of the first cohort of livers were DCD livers, in contrast to just 3 of the second cohort, possibly contributing to the higher incidence in the first cohort.

Post reperfusion syndrome was not reported in the initial pilot study of the Metra (OrganOx, Oxford, UK) NESLiP device ^6^. In that study the livers were placed on the machine at the point of retrieval and suffered little cold ischaemia. In addition the perfusate oxygen tension on Metra is typically between 90 and 150mmHg (12 and 20kPa) (David Nasralla, personal communication). The perfusate composition used in that study was similar to the Gelofusine based perfusate we used.

We used a combination of parameters to assess the livers during perfusion. Prior hepatocellular damage was reflected in the perfusate ALT at 2 hours, by which point most enzyme washout had occurred. Lactate metabolism to glucose or glycogen occurs predominantly in peri-portal hepatocytes, ^38^ so disturbances in lactate metabolism were considered to represent peri-portal hepatocyte damage or ongoing lactate production (eg from poorly perfused parenchyma). Flushing the liver with compound sodium lactate before NESLiP provided a higher baseline lactate whose metabolism could be followed.

Glycogenolysis is an ATP-independent process that continues during cold storage and is enhanced at reperfusion ^39,40^, and explains the raised perfusate glucose seen in many of the NESLiP cases. While a raised glucose is commonly observed during NESLiP, a normal glucose might be a manifestation of glycogen exhaustion and/or extensive lobular damage, or it may signify minimal ischaemia. Hence while a raised perfusate glucose may be a marker of moderate ischaemia, a normal glucose may paradoxically represent either minimal or severe ischaemic damage. This was seen in Case 6, where the glucose was normal, probably as a result of global lobular injury rather than implying a good liver as first thought. In order to rule out severe lobular injury in case 10 a glucose challenge was given after which the glucose fell rapidly. The fall in glucose observed is explained by glucose entry into the liver via the insulin-independent GLUT2 transporter, and its subsequent incorporation into glycogen (data not shown).

Hepatic regulation of acid-base balance depends upon the differential metabolism of glutamine along the lobule ^41^. An inability to regulate pH, with worsening acidosis, was considered to signify pan-lobular hepatocyte damage ^42,43^, and it is noteworthy that case 6 had the greatest tendency to acidosis during perfusion (data not shown).

Bile production has been suggested as a sensitive marker of liver viability during NESLiP ^44-46^. It is a complex process dependent on the integrity of many facets of liver function. Bile acids are secreted predominantly in zones 1 and 2, while bicarbonate is secreted in zone 3 ^47^. Viability of the cholangiocytes will also influence the amount and quality of bile production. Figure 7 shows varying patterns of bile production. Case 10, the poorest bile producer, is a liver with satisfactory function and minimal evidence of cholangiopathy whereas cases 7, and 11 are among the best producers of bile have developed clinically significant cholangiopathy. Case 6, the liver suffering primary non function, also made a reasonable amount of bile. It is unclear from our series how much emphasis should be placed on the amount of bile produced, but the ability to produce an alkali bile might be a more significant marker of cholangiocyte integrity. Where bile was produced and its pH measured, only the livers not able to produce bile with a pH>7.4 went on to develop significant cholangiopathy.

The high incidence of cholangiopathy in this series is in contrast to reports from researchers using cold machine perfusion, albeit of less marginal livers ^48^, but is similar to the contemporaneous non-NESLiP cohort. The presence of severe duct injury visible on duct biopsies before normothermic perfusion in cases 3 and 11 suggests this might be a donor phenomenon and not related to the perfusion technique.

In summary, our report shows that a period of normothermic perfusion before transplantation can allow biochemical assessment of liver function and arrest of cold ischaemia. Hyperoxic perfusates were associated with post reperfusion vasoplegia and haemodynamic instability, possibly as a consequence of release of ROS and RNS, whereas lower perfusate oxygen tensions were associated with an uneventful reperfusion, although the numbers are too small to confidently make a causal association. We noted a high incidence of cholangiopathy which was associated with an inability to produce an alkali pH during NESLiP.

**Acknowledgments**

The research was funded in part by Addenbrooke’s Charitable Trust and in part by the National Institute for Health Research Blood and Transplant Research Unit (NIHR BTRU) in Organ Donation and Transplantation at the University of Cambridge in collaboration with Newcastle University and in partnership with NHS Blood and Transplant (NHSBT). The views expressed are those of the authors and not necessarily those of the NHS, the NIHR, the Department of Health or NHSBT.

We acknowledge the help of our clinical colleagues in performing the transplants and supporting the recipients peri- and postoperatively. We would like to acknowledge the cooperation of Gareth Hayman in helping to facilitate the perfusions that we describe here as well as the many nonclinical perfusions. We also thank Bridget Featherstone for advice regarding perfusate additives, and Marg Negus for assisting with the *in vitro* assays.

**References**

1. Kim WR, Lake JR, Smith JM, et al. OPTN/SRTR 2014 Annual data report: Liver. *Am J Transplant.* 2016;16 Suppl 2: 69-98.

2. NHS Blood and Transplant. Annual Report on Liver Transplantation. Report for 2014/15. <<http://www.odt.nhs.uk/pdf/organ_specific_report_liver_2015.pdf>> Published 2015. Accessed 28th March 2016.

3. NHS Blood and Transplant. Organ donation and Transplantation Activity Report 2014/15. <<http://nhsbtmediaservices.blob.core.windows.net/organ-donation-assets/pdfs/activity_report_2014_15.pdf>> Published 2015. Accessed 28th March 2016.

4. Feng S, Lai JC. Expanded criteria donors. *Clin Liver Dis.* 2014;18(3): 633-649.

5. NHS Blood and Transplant. Policy POL196/4.1 Deceased Donor Liver Distribution and Allocation. <<http://www.odt.nhs.uk/pdf/liver_allocation_policy.pdf>> Published 2015. Accessed 28th March 2016.

6. Ravikumar R, Jassem W, Mergental H, et al. Liver transplantation after ex vivo normothermic machine preservation: a Phase 1 (first-in-man) clinical trial. *Am J Transplant.* 2016;16(6): 1779-1787.

7. Watson CJ, Kosmoliaptsis V, Randle LV, et al. Preimplant Normothermic Liver Perfusion of a Suboptimal Liver Donated After Circulatory Death. *Am J Transplant.* 2016;16(1): 353-357.

8. Aggarwal S, Kang Y, Freeman JA, Fortunato FL, Jr., Pinsky MR. Postreperfusion syndrome: hypotension after reperfusion of the transplanted liver. *J Crit Care.* 1993;8(3): 154-160.

9. de Armas LC, Castillo YA. Is it possible to distinguish between vasoplegic syndrome and postreperfusion syndrome during liver graft reperfusion? *Anesth Analg.* 2010;110(3): 969-970; author reply 970-961.

10. Omar S, Zedan A, Nugent K. Cardiac vasoplegia syndrome: pathophysiology, risk factors and treatment. *Am J Med Sci.* 2015;349(1): 80-88.

11. Valentine E, Gregorits M, Gutsche JT, Al-Ghofaily L, Augoustides JG. Clinical update in liver transplantation. *J Cardiothorac Vasc Anesth.* 2013;27(4): 809-815.

12. Feng S, Goodrich NP, Bragg-Gresham JL, et al. Characteristics Associated with Liver Graft Failure: The Concept of a Donor Risk Index. *Am J Transplant.* 2006;6: 783-790.

13. Schaubel DE, Sima CS, Goodrich NP, Feng S, Merion RM. The survival benefit of deceased donor liver transplantation as a function of candidate disease severity and donor quality. *Am J Transplant.* 2008;8(2): 419-425.

14. Organ Procurement and Transplantation Network. MELD Calculator. <<https://optn.transplant.hrsa.gov/resources/allocation-calculators/meld-calculator/>> Published 2016. Accessed 29 February 2016.

15. NHS Blood and Transplant. UK End-stage liver disease (UKELD) score calculator. <<http://www.odt.nhs.uk/transplantation/guidance-policies/tools/>>. Accessed 28th March 2016.

16. Watson CJ, Randle LV, Kosmoliaptsis V, Gibbs P, Allison M, Butler AJ. 26-hour Storage of a Declined Liver Before Successful Transplantation Using Ex Vivo Normothermic Perfusion. *Ann Surg.* 2016.

17. Mergental H, Perera M, Laing RW, et al. Transplantation of Declined Liver Allografts Following Normothermic Ex-Situ Evaluation. *Am J Transplant.* 2016.

18. Hosgood SA, Nicholson ML. First in man renal transplantation after ex vivo normothermic perfusion. *Transplantation.* 2011;92(7): 735-738.

19. Nicholson ML, Hosgood SA. Renal transplantation after ex vivo normothermic perfusion: the first clinical study. *Am J Transplant.* 2013;13(5): 1246-1252.

20. Hellinger A, Fiegen R, Lange R, et al. Preservation of pig liver allografts after warm ischemia: normothermic perfusion versus cold storage. *Langenbecks Arch Chir.* 1997;382(4): 175-184.

21. Liu Q, Nassar A, Farias K, et al. Sanguineous normothermic machine perfusion improves hemodynamics and biliary epithelial regeneration in donation after cardiac death porcine livers. *Liver Transpl.* 2014;20(8): 987-999.

22. Op den Dries S, Karimian N, Porte RJ. Normothermic machine perfusion of discarded liver grafts. *Am J Transplant.* 2013;13(9): 2504.

23. op den Dries S, Karimian N, Sutton ME, et al. Ex vivo normothermic machine perfusion and viability testing of discarded human donor livers. *Am J Transplant.* 2013;13(5): 1327-1335.

24. Banan B, Watson R, Xu M, Lin Y, Chapman W. Development of a Normothermic Ex-vivo Liver Perfusion (NELP) System towards Improving Viability and Function of Human Extended Criteria Donor livers. *Liver Transpl.* 2016.

25. Schon MR, Kollmar O, Wolf S, et al. Liver transplantation after organ preservation with normothermic extracorporeal perfusion. *Ann Surg.* 2001;233(1): 114-123.

26. Murphy MP. How mitochondria produce reactive oxygen species. *Biochem J.* 2009;417(1): 1-13.

27. van Golen RF, Reiniers MJ, Vrisekoop N, et al. The mechanisms and physiological relevance of glycocalyx degradation in hepatic ischemia/reperfusion injury. *Antioxid Redox Signal.* 2014;21(7): 1098-1118.

28. van Golen RF, van Gulik TM, Heger M. Mechanistic overview of reactive species-induced degradation of the endothelial glycocalyx during hepatic ischemia/reperfusion injury. *Free Radic Biol Med.* 2012;52(8): 1382-1402.

29. Joachimsson PO, Sjoberg F, Forsman M, Johansson M, Ahn HC, Rutberg H. Adverse effects of hyperoxemia during cardiopulmonary bypass. *J Thorac Cardiovasc Surg.* 1996;112(3): 812-819.

30. Ihnken K, Winkler A, Schlensak C, et al. Normoxic cardiopulmonary bypass reduces oxidative myocardial damage and nitric oxide during cardiac operations in the adult. *J Thorac Cardiovasc Surg.* 1998;116(2): 327-334.

31. Spoelstra-de Man AM, Smit B, Oudemans-van Straaten HM, Smulders YM. Cardiovascular effects of hyperoxia during and after cardiac surgery. *Anaesthesia.* 2015;70(11): 1307-1319.

32. Hayes RA, Shekar K, Fraser JF. Is hyperoxaemia helping or hurting patients during extracorporeal membrane oxygenation? Review of a complex problem. *Perfusion.* 2013;28(3): 184-193.

33. Ezaki S, Suzuki K, Kurishima C, et al. Resuscitation of preterm infants with reduced oxygen results in less oxidative stress than resuscitation with 100% oxygen. *J Clin Biochem Nutr.* 2009;44(1): 111-118.

34. Zinchuk VV, Khodosovsky MN, Maslakov DA. Influence of different oxygen modes on the blood oxygen transport and prooxidant-antioxidant status during hepatic ischemia/reperfusion. *Physiol Res.* 2003;52(5): 533-544.

35. Serviddio G, Di Venosa N, Federici A, et al. Brief hypoxia before normoxic reperfusion (postconditioning) protects the heart against ischemia-reperfusion injury by preventing mitochondria peroxyde production and glutathione depletion. *FASEB J.* 2005;19(3): 354-361.

36. Martell M, Coll M, Ezkurdia N, Raurell I, Genesca J. Physiopathology of splanchnic vasodilation in portal hypertension. *World J Hepatol.* 2010;2(6): 208-220.

37. Morita K. Surgical reoxygenation injury of the myocardium in cyanotic patients: clinical relevance and therapeutic strategies by normoxic management during cardiopulmonary bypass. *Gen Thorac Cardiovasc Surg.* 2012;60(9): 549-556.

38. Dancygier H. Functional heterogeneity and metabolic zonation. In: Dancygier H, ed. *Clinical hepatology: Principles and practice of hepatobiliary diseases*. Berlin Heidelberg: Springer-Verlag; 2010.

39. Cherid A, Cherid N, Chamlian V, et al. Evaluation of glycogen loss in human liver transplants. Histochemical zonation of glycogen loss in cold ischemia and reperfusion. *Cell Mol Biol (Noisy-le-grand).* 2003;49(4): 509-514.

40. Dodero F, Benkoel L, Allasia C, et al. Quantitative analysis of glycogen content in hepatocytes of human liver allograft after ischemia and reperfusion. *Cell Mol Biol (Noisy-le-grand).* 2000;46(7): 1157-1161.

41. Brosnan ME, Brosnan JT. Hepatic glutamate metabolism: a tale of 2 hepatocytes. *Am J Clin Nutr.* 2009;90(3): 857S-861S.

42. Atkinson DE, Camien MN. The role of urea synthesis in the removal of metabolic bicarbonate and the regulation of blood pH. *Curr Top Cell Regul.* 1982;21: 261-302.

43. Häussinger D. Liver and kidney in acid-base regulation. *Nephrol Dial Transplant.* 1995;10(9): 1536.

44. Bowers BA, Branum GD, Rotolo FS, Watters CR, Meyers WC. Bile flow--an index of ischemic injury. *J Surg Res.* 1987;42(5): 565-569.

45. Imber CJ, St Peter SD, de Cenarruzabeitia IL, et al. Optimisation of bile production during normothermic preservation of porcine livers. *Am J Transplant* 2002;2(7): 593-599.

46. Sutton ME, op den Dries S, Karimian N, et al. Criteria for viability assessment of discarded human donor livers during ex vivo normothermic machine perfusion. *PLoS ONE.* 2014;9(11): e110642.

47. Gebhardt R. Metabolic zonation of the liver: regulation and implications for liver function. *Pharmacol Ther.* 1992;53(3): 275-354.

48. Dutkowski P, Polak WG, Muiesan P, et al. First Comparison of Hypothermic Oxygenated PErfusion Versus Static Cold Storage of Human Donation After Cardiac Death Liver Transplants: An International-matched Case Analysis. *Ann Surg.* 2015;262(5): 764-770; discussion 770-761.

49. Collett D, Friend PJ, Watson CJE. Factors associated with short and long term liver graft survival in the United Kingdom: development of a UK Donor Liver Index. [published online ahead of print December 1, 2016] *Transplantation.* DOI: 10.1097/TP.0000000000001576.

50. Ali JM, Davies SE, Brais RJ, et al. Analysis of ischemia/reperfusion injury in time-zero biopsies predicts liver allograft outcomes. *Liver Transpl.* 2015;21(4): 487-499.

51. op den Dries S, Westerkamp AC, Karimian N, et al. Injury to peribiliary glands and vascular plexus before liver transplantation predicts formation of non-anastomotic biliary strictures. *J Hepatol.* 2014;60(6): 1172-1179.

52. Hansen T, Hollemann D, Pitton MB, et al. Histological examination and evaluation of donor bile ducts received during orthotopic liver transplantation--a morphological clue to ischemic-type biliary lesion? *Virchows Arch.* 2012;461(1): 41-48.

**Table 1. Donor and perfusion details**

| **Case no.** | **Donor** | | | | | | | | **Perfusion** | | | | | | | |
| --- | --- | --- | --- | --- | --- | --- | --- | --- | --- | --- | --- | --- | --- | --- | --- | --- |
|  | **Age** | **Offer type^1^** | **DCD / DBD** | **Agonal period (mins) ^2^** | **Asystolic period (mins)^3^** | **Cause of death** | **US DRI^4^** | **UK DLI** | **Pre-NESLiP cold ischaemia (mins)** | **NESLiP duration (mins)** | **Total  ex situ storage (mins)^5^** | **Mean arterial pO_2_ during perfusion** | | **Mean flows during NESLiP** | | **Perfusate ALT at 2 hours (iu/L)** |
|  |  |  |  |  |  |  |  |  |  |  |  | **kPa** | **mmHg** | **HA (ml/min)** | **PV (L/min)** |  |
| **1** | 57 | Zonal | DCD | 150 | 10 | Head injury | 2.01 | 2.27 | 360 | 132 | 572 | 83 | 621 | 260 | 0.82 | 1305 |
| **2** | 24 | National | DCD | 5 | 12 | Hypoxia | 1.92 | 1.34 | 419 | 321 | 790 | 88 | 659 | 319 | 1.13 | 906 |
| **3** | 57 | Zonal | DCD | 30 | 11 | Intracranial haemorrhage | 2.09 | 2.11 | 346 | 262 | 681 | 86 | 646 | 233 | 0.93 | 2373 |
| **4** | 63 | Zonal | DCD | 20 | 11 | Head injury | 2.08 | 2.00 | 222 | 272 | 564 | 86 | 647 | 208 | 1.03 | 1478 |
| **5** | 45 | National | DCD | 17 | 14 | Head injury | 2.12 | 1.44 | 445 | 261 | 765 | 85 | 641 | 390 | 0.97 | 3783^5^ |
| **6** | 48 | Fast track | DCD | 22 | 14 | Hypoxia | 2.45 | 1.92 | 438 | 491 | 1039 | 90 | 671 | 236 | 0.99 | 9490 |
| **7** | 55 | Zonal | DCD | 19 | 11 | Intracranial haemorrhage | 2.51 | 1.58 | 396 | 295 | 742 | 21 | 154 | 239 | 0.96 | 2283 |
| **8** | 60 | National | DBD | 0 | 0 | Intracranial haemorrhage | 2.17 | 0.80 | 608 | 459 | 1136 | 24 | 176 | 219 | 1.09 | 5576 |
| **9** | 58 | Research | DCD | 32 | 5 | Intracranial haemorrhage | 3.14 | 1.97 | 389 | 436 | 897 | 25 | 187 | 285 | 0.73 | 1118 |
| **10** | 39 | Research | DBD | 0 | 0 | Hypoxia | 1.47 | 0.86 | 618 | 530 | 1561 | 25 | 186 | 210 | 0.75 | 418 |
| **11** | 54 | Fast track | DCD | 12 | 12 | Hypoxia | 2.81 | 2.83 | 435 | 163 | 665 | 23 | 173 | 261 | 0.68 | 913 |
| **12** | 67 | Fast track | DBD | 0 | 0 | Hypoxia | 2.44 | 1.66 | 877 | 122 | 1071 | 20 | 153 | 349 | 0.66 | 555 |

1. Liver offers were from donors within the recipient center’s allocation zone (“Zonal”), or elective offers from outside of zone (“National”) where the zonal center declined the liver, or fast track offers where the liver was offered after retrieval as part of the UK fast track scheme (“Fast track”). Where the liver has been turned down by all centers it is then offered for research.
2. Agonal period defined as time from withdrawal of treatment to circulatory arrest in donor.
3. Asystolic period, also known as secondary warm ischaemic time, from circulatory arrest to cold *in situ* perfusion.
4. US Donor Risk Index ^12,13^: In calculating this the cold ischaemic time taken up to the point of commencing normothermic perfusion. A DRI >2.0 was associated with a 3-month graft survival ≤80% in the original description of the index.
5. UK Donor Liver Index^49^: Unlike the US Donor Risk Index, the UK DLI is based on UK data and is an index of liver quality at the point of donation and does not consider ischaemic time. An index > 1.24 represents the livers with a 65% higher chance of graft failure than livers with a DLI<1.24.
6. Total *ex situ* storage, includes period from end of normothermic perfusion to reperfusion in the recipient (portal first in all cases).
7. Value is at one hour post perfusion.

**Table 2. Recipient details.**

| **Case No** | **Age** | **Diagnosis** | **MELD / UKELD^1^** | **Potassium pre / post reperfusion** (mmol/L)^2^ | **Post reperfusion syndrome  (% pre-MAP)^3^** | **Post reperfusion vasoplegia^4^** | **Inotrope requirement before and after reperfusion** | | **Comments** |
| --- | --- | --- | --- | --- | --- | --- | --- | --- | --- |
|  |  |  |  |  |  |  | **Prereperfusion** | **Postreperfusion** |  |
| **1** | 58 | ALD | 14 / 50 | 5.0 / 5.4 | 68 / 85 = 85% | No | Nil | Metaraminol boluses to 1.5mg | Liver described as steatotic by retrieving surgeon but appeared normal on arrival at recipient centre. |
| **2** | 57 | ALD | 16 / 55 | 4.0 / 4.1 | 49 / 72 = 69% | No | NorEpi 0.19µg/kg/min;  Metaraminol boluses to 1mg | NorEpi increased to 0.24 to 0.27µg/kg/min;  Metaraminol boluses to 2mg  MAP ≤54mmHg for 80mins | Pulmonary thromboembolism during explant requiring inotrope support; further emboli occurred during posttransplant course. |
| **3** | 46 | ALD and a1AT | 17 / 57 | 4.6 / 4.4 | 37 / 60 = 62% | Yes | NorEpi 0.05µg/kg/min | NorEpi increased to 0.10µg/kg/min  Metaraminol boluses to 1mg.  Epinephrine boluses to 150µg.  Argipressin started 4h later.  MAP<50mmHg for 35 minutes  Cardiac index 9.9 |  |
| **4** | 65 | ALD | 26 / 64 | 4.6 / 4.9 | 43 / 61 = 70% | Yes | NorEpi 0.1µg/kg/min | NorEpi 0.10µg/kg/min  Metaraminol boluses to 1mg  Epinephrine boluses to 450µg, then infusion of 0.1µg/kg/min  MAP 40 – 60 for 75 minutes.  Cardiac index 2.7 | Coagulopathy and subcapsular haematomas requiring peri-hepatic packing. |
| **5** | 65 | a1AT | 17 / 54 | 4.5 / 4.5 | 49 / 72 = 68% | Yes | Metaraminol 10mg/h | Ephedrine 6mg  Metaraminol boluses to 2mg.  Epinephrine 30µg in first 30min  Argipressin 20u/h infusion  MAP <50mmHg for 40min | Laceration segment 6 of liver at time of traumatic donor death. |
| **6** | 59 | NAFLD / HCC | 25 / 62 | 5.7 / 6.3 | 42 / 65 = 64% | Yes | NorEpi 0.04µg/kg/min | NorEpi 0.10 – 0.15µg/kg/min in first 60 mins, then  Argipressin 10u/h.  Epinephrine 0.1-0.5µg/kg/min added from 2 hours postreperfusion  MAP 40-70 for 3 hours post reperfusion | 28 litre blood loss during explant and before implantation of new liver began, 51L in total. Primary non function; recipient died 48 hours later despite urgent retransplant. |
| **7** | 63 | CVID | 10 / 52 | 4.2 / 4.8 | 52 / 62 = 84% | No | Metaraminol 20mg/h | Metaraminol boluses to 30mg, infusion increased to 50mg/h | Liver described as moderately steatotic by retrieving surgeon but minimal on inspection by recipient surgeon and on histology. |
| **8** | 49 | a1AT / NAFLD / HCC | 11 / 52 | 4.0 / 5.4 | 50 / 68 = 74% | No | Metaraminol boluses to 4mg | Metaraminol boluses to 7mg  NorEpi infusion 0.10 µg/kg/min | Donor liver contained 30-60% macrovesicular fat on histology. At closure developed subcapsular haematomas requiring peri-hepatic packing for 48 hours. |
| **9** | 63 | HCC and HCV | 11 / 50 | 3.9 / 5.5 | 70 / 80 = 88% | No | Metaraminol boluses to 5mg | Metaraminol boluses to 3mg;  NorEpi infusion 0.08µg/kg/min | Laceration and haematoma in left lateral segment at time of death managed with fibrin glue and topical haemostat. |
| **10** | 49 | PSC | 26 / 63 | 5.0 / 4.0 | 51 / 65 = 78% | No | Nil | metaraminol bolus 1mg | Delayed implant due to suspicious lesion found on liver capsule requiring frozen section histology to exclude malignancy. |
| **11** | 57 | a1AT / NAFLD | 25 / 59 | 5.1 / 4.4 | 53 / 68 = 78% | No | Metaraminol 5mg/h | Metaraminol Infusion unchanged.  No additional vasopressor |  |
| **12** | 48 | Auto-immune hepatitis | 13 / 49 | 3.9 / 4.9 | 63 / 75 = 84% | No | Metaraminol 5mg/h | Metaraminol 1mg bolus. Infusion unchanged.  No additional vasopressor | RHA divided in donor requiring reconstruction before NESLiP. Late change in recipient resulting in long period of cold storage. |

1. The Model for end-stage liver disease score (MELD) was calculated from the Organ Procurement and Transplantation Network (OPTN) website ^14^ and the UK End stage liver disease (UKELD) score from the National Health Service Blood and Transplant (NHSBT) website ^15^ . In the UK, a UKELD score >49 corresponds to a better survival with a transplant than remaining on the waiting list.
2. Potassium recorded immediately before reperfusion during anhepatic phase and within 5 minutes following reperfusion
3. Post reperfusion syndrome defined as a fall in mean arterial pressure (MAP) within 5 minutes of reperfusion to ≤ 70% of the baseline value in the last 5 minutes of the anhepatic period ^8^. The numbers represent the MAP in the anhepatic period and the lowest in the first 5 minutes postreperfusion.
4. Vasoplegia was defined as a fall in mean arterial pressure (MAP) to < 50 mmHg *either* sustained for >30 mins in spite of pressors *or* requiring high doses of pressors (defined as norepinephrine >0.15µg/kg/min, argipressin >2u/hr, or infusion of epinephrine).

Abbreviations: ALD: Alcohol related liver disease; a1AT: alpha-1 anti-trypsin deficiency; NAFLD: Nonalcoholic fatty liver disease; CVID: combined variable immunodeficiency; HCC: hepatocellular carcinoma; HCV: Hepatitis C cirrhosis; PSC: Primary sclerosing cholangitis; IC Ischaemic cholangiopathy; NorEpi: norepinephrine; MAP: mean arterial pressure

**Table 3. Histological findings of liver and bile duct before and after NESLiP**

|  | **Liver ^1^** | | **Bile duct Injury^2^** | | **pH of bile during NESLiP** | **MRCP** |
| --- | --- | --- | --- | --- | --- | --- |
| **Case** | **Preimplant^3^** | **Postreperfusion in recipient** | **Preimplant^3^** | **Postreperfusion in recipient** |  |  |
| **1** | No biopsy | Steatosis: none  Necrosis: none  Reperfusion injury: minimal | No biopsy | **Grade 2 injury.**  Stroma 25-50% necrotic  Periluminal PBG >50% loss  Deep PBG <50% loss | No bile collected | Normal at 6mo |
| **2** | No biopsy | Steatosis: none  Necrosis: none  Reperfusion injury: mild | No biopsy | No biopsy | No bile collected | Normal at 6mo |
| **3** | Biopsy pre-NESLiP:  Steatosis: none  Necrosis: none | Steatosis: none  Necrosis: patchy and severe, zones 2 and 3, portal stroma and arterioles, degenerate ducts  Reperfusion injury: moderate | Biopsy pre-NESLiP  **Grade 2 injury**  Stroma 25-50% necrotic  Periluminal PBG >50% loss  Deep PBG > 50% loss | **Grade 3 injury**  Stroma > 50% necrotic  Periluminal PBG lost  Deep PBG lost.  No viable stroma present. | Not measured | Cholangiopathy confirmed at 7mo. |
| **4** | Biopsy pre-NESLiP:  Steatosis: mild with steatohepatitis in zone 3  Necrosis: none | Steatosis: mild  Necrosis: none  Reperfusion injury: mild | Biopsy pre-NESLiP  **Grade 2 injury**  Stroma 25-50% necrotic  Periluminal PBG >50% loss  Deep PBG > 50% loss | **Grade 2 injury**  Stroma 25-50% necrotic  Periluminal PBG >50% loss  Deep PBG <50% loss | Not measured | Normal at 4mo |
| **5** | Biopsy post NESLiP  Steatosis: <5%  Necrosis: none | Steatosis: < 5%  Necrosis: panlobular  Reperfusion injury: severe | Biopsy post-NESLiP  **Grade 2 injury**  Stroma 25-50% necrotic  Periluminal PBG > 50% loss  Deep PBG >50% loss | **Grade 1**  Stroma <25% necrotic  Periluminal PBG <50% loss  Deep PBG <50% loss | pH 7.6 | Normal at 6mo |
| **6** | No biopsy | Histological examination of explanted failed transplant showed global parenchymal ischaemic necrosis with preservation of a rim of cells around the central veins | No biopsy | Grade 2 injury (biopsy of hilar duct of explanted transplant)  Periluminal PBG >50% loss  Deep PBG >50% loss | pH 7.5 | Died day 2 |
| **7** | Biopsy pre-NESLiP  Steatosis: <5%  Necrosis: none | Steatosis: < 5%  Necrosis: patchy, severe, confluent zones 2 and 3  Reperfusion injury: moderate | No biopsy | No biopsy | pH 6.9 | Cholangiopathy confirmed at 2mo. |
| **8** | No biopsy | Steatosis: moderate  Necrosis: none  Reperfusion injury: mild | No biopsy | **Grade 3**  Stroma > 50% necrotic  Periluminal PBG lost  Deep PBG lost | pH 7.4-7.8 | Normal at 8 months |
| **9** | Biopsy post NESLiP  Steatosis: none  Necrosis: scattered acidophil bodies  Reperfusion injury: mild to moderate | Steatosis: none  Necrosis: zones 2 and 3 early coagulative necrosis  Reperfusion injury: severe | Biopsy post-NESLiP  **Grade 1**  Stroma <25% necrotic  Periluminal PBG < 50% loss  Deep PBG < 50% loss | **Grade 2**  Stroma 25-50% necrotic  Periluminal PBG >50% loss  Deep PBG <50% loss | pH 7.5 | Normal at 6 months |
| **10** | Biopsy pre-NESLiP  Steatosis: none  Necrosis: none  Reperfusion injury: none | No biopsy | Biopsy post-NESLiP  **Grade 2**  Stroma 25-50% necrotic  Periluminal PBG > 50% loss  Deep PBG >50% loss | No biopsy | pH 7.7 | Mild duct irregularity at 6 months. Normal liver biochemistry |
| **11** | Biopsy pre-NESLiP  Steatosis: none  Necrosis: none  Reperfusion injury: none  Other findings: Several mitotically active cells | Steatosis: none  Necrosis: none  Reperfusion injury: mild  Other findings: mitotically active cells, necrotic septal duct.  (Biopsy was post-NESLiP, preimplant) | Biopsy pre-NESLiP  **Grade 3**  Stroma 25- 50% necrotic  Periluminal PBG lost  Deep PBG lost  Biopsy post-NESLiP  **Grade 3 (worse)**  Stroma >50% necrotic  Periluminal PBG lost  Deep PBG lost | No biopsy | pH 7.2 | Cholangiopathy confirmed at 2mo. Retransplant at 6 months. |
| **12** | No biopsy | Steatosis: none  Necrosis: none  Reperfusion injury: minimal | No biopsy | **Grade 1**  Stroma <25% necrotic  Periluminal PBG <50% loss  Deep PBG <50% loss | No bile collected | Normal at 6 months |

1) Reperfusion injury is graded as nil, mild, moderate or severe; steatosis is graded none, (<5% of hepatocytes); mild (5-30%); moderate (30-60%) after Ali et al. ^50^.

2) Bile duct histology is given an overall grade of injury after the system of op den Dries ^51^ and Hansen ^52^, which looks at the integrity of biliary epithelium, mural stromal necrosis, damage to the peribiliary arteriolar plexus, thrombus within the plexus, extent of intramural haemorrhage, integrity of the peri-luminal and deep peribiliary glands (PBG), and presence of inflammation. Changes affecting the stroma and periluminal and deep peribiliary glands are highlighted. Extensive (>50%) damage to the superficial biliary epithelium was present in nearly all ducts examined and may have reflected injury by the cannula placed in the duct as well as relating to ischaemic damage. Similarly, there was peribiliary vascular plexus injury and no thrombosis seen.

3) Preimplant biopsies were taken either before NESLiP or after; Post reperfusion biopsies were taken immediately prior to the biliary anastomosis, in the case of the bile duct, and at the end of the transplant immediately prior to abdominal closure in the case of the liver biopsy.

**Table 4. Outcomes of livers subject to NESLiP compared to a cohort of contemporaneous liver transplants.**

|  | **Hyperoxic NESLiP** | **Normoxic NESLiP** | **All NESLiP** | **Comparator cohort** |
| --- | --- | --- | --- | --- |
| Number of livers | 6 | 6 | 12 | 24 |
| Offer type: Fast track offers  Zonal offers  National offers  Research offers | 1  3  2  0 | 2  1  1  2 | 3  4  3  2 | 4  16  4  0 |
| Donor type: DBD  DCD | 0  6 | 3  3 | 3  9 | 6  18 |
| Donor age (median (range)) | 53 (24-62) | 56 (39-67) | 56 (24-67) | 54 (22-72) |
| Time from withdrawal to cold perfusion for DCDs, mins (median (range) | 34 (17 – 160) | 30 (24-34) | 31 (17 – 160) | 22 (12 – 124) |
| Cold ischaemic time, mins (median (range)) | 390 (222-445) | 522 (389-877) | 427 (222-877) | 439 (333 – 720) |
| NESLiP duration, mins (median (range)) | 267 (132 – 491) | 365 (122 – 530) | 284 ( 122 – 530) | - |
| Extracorporeal storage time, mins (median (range)) | 723 (564-1039) | 984 (665 – 1561) | 777 (564 – 1561) | 439 (333 – 720) |
| US Donor Risk Index (median (range)) ^12^ | 2.1 (1.9 – 2.5) | 2.5 (1.5 - 3.1) | 2.1 (1.5 – 3.1) | 2.2 (1.5 – 3.8) |
| UK Donor Liver index (median (range)) ^49^ | 2.0 (1.3 – 2.3) | 1.6 (0.8 – 2.8) | 1.8 (0.8 – 2.8) | 2.0 (1.0 – 2.9) |
| Post reperfusion syndrome incidence | 5/6 = 83% | 0/6 = 0% | - | 6/24 = 25% |
| Vasoplegia incidence | 4/6 = 67% | 0/6 = 0% | - | 5/24 = 21% |
| Peak ALT in first 7 days, iu/L (median (range)) | 1210 (329 - 4991) | 780 (187 – 1743) | 1069 (187 – 4991) | 787 (155 – 2238) |
| Cholangiopathy (ignoring patient who died day 2) | 1/5 = 20% | 2/6 = 33% | 3/11 = 27% | 7/24 = 29% |
| Actual graft survival (median follow up 12 months, range 9 -24) | 5/6 = 83% | 5/6= 83% | 10/12 = 83% | 21/24 = 88% |
| Actual patient survival (median follow up 12 months, range 9 -24) | 5/6= 83% | 6/6 = 100% | 11/12 = 92% | 23/24 = 96% |

**Figure Legends**

**Figure 1. Changes in protocol for Normothermic Ex Situ Liver Perfusion**

Comparison of the protocols for flushing and perfusing the first and last 6 livers undergoing NESLiP

**Figure 2. Storage times of the 12 livers, broken down by periods of storage**

Horizontal bars represent individual livers, showing the time periods from withdrawal of treatment, asystole, cold storage, and normothermic perfusion, removal from the machine and being cold flushed during implantation, up to reperfusion in the recipient.

**Figure 3. Perfusate glucose concentration during normothermic *ex situ* liver perfusions**

Individual lines represent the change in perfusate glucose for each liver in the series; blue lines with open symbols represent livers perfused with low oxygen tensions. With 3 exceptions there was a release of glucose on reperfusion of the liver followed by a slow fall towards normal. Note the glucose in livers 3, 6 and 10 were in the “normal” range initially. Case 10 received an infusion of glucose between 4 and 6 hours, following which there was a spontaneous and rapid fall.

**Figure 4. Perfusate lactate concentration during normothermic *ex situ* liver perfusion**

Lactate concentrations for each liver in the series. Note the spontaneous fall in all cases. The livers in the last 6 cases were flushed with compound sodium lactate (Hartmann’s solution) before perfusion, washing out potassium and loading the liver with lactate to enable more ready assessment of a fall. Cases 5 and 9 were livers that had suffered a degree of parenchymal trauma; the delayed lactate fall in 9 and incomplete fall in 5 were interpreted in that light, with presumed on going lactate production in the damaged segments. Case 6, the slowest fall, suffered primary non function.

**Figure 5. Perfusate ALT during normothermic *ex situ* liver perfusion**

Perfusate ALTs for each liver during perfusion. Case 6 suffered primary nonfunction, and case 8 was a steatotic liver.

**Figure 6. Relationship between perfusate ALT after 2 hours and the peak ALT posttransplantation.**

The peak ALT in the first 7 days posttransplant is plotted against the perfusate ALT after 2 hours. Note case 6 developed primary nonfunction. There was a significant correlation between the values (correlation coefficient R^2^=0.56, p=0.005). The dotted line is a liner regression plot constrained through the origin.

**Figure 7. Cumulative bile production during normothermic *ex* situ liver perfusion.**

Bile production varied, and did not predict cholangiopathy or viability. Note that cases 7 and 11 have evidence of ischaemic cholangiopathy on MRCP even though they had some of the highest rates of bile production; they also had the least alkali bile. Bile production could not be recorded in 3 livers due to occlusion of the biliary catheter. Bile salts were not added to the perfusate.

**Figure 8. Posttransplant biochemistry**

(8a) Posttransplant ALT (Normal range <50iu/L). The ALT fell to normal in all patients except case 6 (not shown). The highest ALTs were in the 2 cases with parenchymal lacerations at the time of donation (cases 5 and 9).
(8b) Posttransplant ALP (normal range <135iu/L). Cases 3, 4 and 7 have persistently raised ALP posttransplant. Intrahepatic biliary strictures have been demonstrated by MRCP in cases 3, 7, and 11. Case 4 has a 6cm hilar mass in conjunction with a persistently positive EBV PCR; he also had an anastomotic biliary stricture dilated 300days posttransplant, although the relationship of this to the hilar mass is unclear.

(8c). Posttransplant prothrombin time. The prothrombin time was persistently raised in case 12 with no obvious cause or clinical consequence. Case 2 was on warfarin for a prosthetic aortic valve, although post operatively she was maintained initially on subcutaneous low molecular weight heparin.

(8d). Posttransplant bilirubin. The bilirubin is raised in those cases with cholangiopathy (3, 7, and 11).
